# CRISPR/Cas9 system is a suitable gene targeting editing tool to filamentous fungus *Monascus pilosus*

**DOI:** 10.1007/s00253-023-12865-x

**Published:** 2024-01-19

**Authors:** Yunxia Gong, Shengfa Li, Qianrui Liu, Fusheng Chen, Yanchun Shao

**Affiliations:** 1 College of Food Science and Technology, Huazhong Agricultural University, Wuhan, 430070 China; 2 Hubei International Scientific and Technological Cooperation Base of Traditional Fermented Foods, Huazhong Agricultural University, Wuhan, 430070 China

**Keywords:** *Monascus* species, *Agrobacterium tumefaciens*-mediated transformation, CRISPR/Cas9 system, Gene replacement frequency, DNA damage response, MK-producing ability

## Abstract

**Abstract:**

*Monascus pilosus* has been used to produce lipid-lowering drugs rich in monacolin K (MK) for a long period. Genome mining reveals there are still many potential genes worth to be explored in this fungus. Thereby, efficient genetic manipulation tools will greatly accelerate this progress. In this study, we firstly developed the protocol to prepare protoplasts for recipient of CRISPR/Cas9 system. Subsequently, the vector and donor DNA were co-transformed into recipients (10^6^ protoplasts/mL) to produce 60–80 transformants for one test. Three genes (*mpclr4*, *mpdot1*, and *mplig4*) related to DNA damage response (DDR) were selected to compare the gene replacement frequencies (GRFs) of *Agrobacterium tumefaciens*-mediated transformation (ATMT) and CRISPR/Cas9 gene editing system (CGES) in *M. pilosus* MS-1. The results revealed that GRF of CGES was approximately five times greater than that of ATMT, suggesting that CGES was superior to ATMT as a targeting gene editing tool in *M. pilosus* MS-1. The inactivation of *mpclr4* promoted DDR via the non-homologous end-joining (NHEJ) and increased the tolerances to DNA damaging agents. The inactivation of *mpdot1* blocked DDR and led to the reduced tolerances to DNA damaging agents. The inactivation of *mplig4* mainly blocked the NHEJ pathway and led to obviously reduced tolerances to DNA damaging agents. The submerged fermentation showed that the ability to produce MK in strain Δ*mpclr4* was improved by 52.6% compared to the wild type. This study provides an idea for more effective exploration of gene functions in *Monascus* strains.

**Key points:**

• *A protocol of high-quality protoplasts for CGES has been developed in M. pilosus.*

• *The GRF of CGES was about five times that of ATMT in M. pilosus.*

• *The yield of MK for Δmpclr4 was enhanced by 52.6% compared with the wild type.*

**Supplementary Information:**

The online version contains supplementary material available at 10.1007/s00253-023-12865-x.

## Introduction

*Monascus* spp. are important industrial fungi widely applied to food and pharmaceutical fields. Traditionally, *Monascus* spp*.* are the producers of red yeast rice (RYR, also called Hongqu), which have been used as food colorant, food preservative, and folk medicine (Lin et al. 2008). With the development of modern science, some beneficial compounds such as *Monascus* pigments (MPs), monacolin K (MK), and γ-aminobutyric acid (GABA) have been identified (Higa et al. 2020; Patakova 2013). However, genome data suggested that there are plenty of backbone genes encoding secondary metabolites (SMs) with unknown functions (He et al. 2018). Therefore, the efficient genetic manipulation tools are required for mining gene information. There are many established genetic manipulation methods that can introduce exogenous DNA into the cells of filamentous fungi, mainly including protoplast-mediated transformation (PMT), lithium acetate-mediated transformation (LAMT), electroporation (EP), biolistic transformation (BT), shock wave-mediated transformation (SWMT), and *Agrobacterium tumefaciens*-mediated transformation (ATMT) (Li et al. 2017). For the simple operation process, ATMT is the most commonly used genetic manipulation method in *Monascus* spp. (Shao et al. 2014). However, due to the non-homologous end joining (NHEJ) pathway of the wild type (WT), the gene replacement frequencies (GRFs) of ATMT in *Monascus* spp. remain relatively low. While He et al. (2013, 2014) improved the GRF of *Monascus ruber* M7 to some extent by inactivating the NHEJ pathway, the modified hosts only allow limited genetic manipulation due to very few available screening markers in *Monascus* spp., which makes it difficult to study the functions of multiple genes.

The “Clustered Regularly Interspaced Short Palindromic Repeats” (CRISPR)/CRISPR-associated protein 9 (Cas9) is a nascent gene editing method. Nødvig et al. (2015) designed a versatile CRISPR/Cas9 gene editing system (CGES) for genetic engineering of filamentous fungi by modifying Cas protein and single-guide RNA (sgRNA), which greatly promoted the application of CGES in filamentous fungi. The typical advantage of this versatile CGES is the high efficiency of targeted gene editing, easier elimination of selection markers free from chromosomes, and broad industrial applicability. For instance, CGES combined with donor DNA fragments has been applied to knock out multiple genes in *Aspergillus niger*, and the GRF of this system was as high as 90% (Leeuwe et al. 2019). Although CGES has been successfully applied to *Monascus purpureus* (Liu et al. 2022) and *M. ruber* (Ree et al. 2023), there are more than 10 species recognized internationally in genus *Monascus*, and they are interspecific diversities with different characteristics (He et al. 2018), so the GRF of CGES in other *Monascus* species remains to be verified.

During the fermentation process, *Monascus* spp. have to cope with various environmental stresses, such as limited nutrients and oxygen supply, produced reactive oxygen species (ROS), and osmolarity (Zeng et al. 2018). These environmental changes may lead to DNA damage and genetic instability, and trigger a response for DNA repair factors and cell cycle regulatory proteins contributing to DNA damage repair (DDR) (Aguilera and Gómez-González 2008). In this study, ATMT and CGES were applied to the strain MS-1 (a high MK-producing strain) for inactivating three genes including *mpclr4* (necessary for pericentromeric formation) (Hall et al. 2002), *mpdot1* (essential for telomere stability) (Nguyen and Zhang 2011), and *mplig4* (responsible for DNA repair) (He et al. 2014). On these foundations, the tolerances of these transformants to different DNA damaging agents were detected to test their effects on DDR. Besides, the capacity of producing MK for these transformants was evaluated by submerged fermentation. Comparison of the genetic transformation efficiencies (GTEs) and GRFs of ATMT and CGES suggests that ATMT is more suitable for constructing random insertion libraries, while CGES is more conducive to perform site-specific gene editing in *Monascus pilosus*.

## Materials and methods

### Plasmids, fungal strain, media, and growth conditions

*M. pilosus* MS-1 (CCTCC M 2013295, China Center for Type Culture Collection (CCTCC), Wuhan, China) with a high yield of MK was used as the WT (Feng et al. 2016). For vector propagation, *Escherichia coli* DH5α (TransGen, Beijing, China) was cultured at 37 °C in Luria-Bertani (LB) broth plus kanamycin or ampicillin (50 μg/mL). *A. tumefaciens* EHA105 was cultured at 28 °C in LB broth plus kanamycin (50 μg/mL) for transformation. An *hph* expression cassette was amplified from plasmid pSKH as a selectable marker (Shao et al. 2017). Plasmid pCAMBIA3300 was the carrier of knockout cassette in ATMT (He et al. 2013). Plasmids pFC332 and pFC334 were used for vector construction in CGES (Nødvig et al. 2015). For observation of colony morphology, PDA, MA (1 L of 15 °Bx wort and 15 g agar), CYA (0.3% NaNO_3_, 0.1% K_2_HPO_4_, 0.05% KCl, 0.05% MgSO_4_·7H_2_O, 0.001% FeSO_4_·7H_2_O, 0.5% yeast extract, 3% sucrose, and 1.5% agar), and G25N (CYA medium containing 25% glycerol (v/v)) were prepared.

### Sequence analysis by bioinformatics prediction

Based on the genome information of strain MS-1 sequenced by PacBio Sequel platform (PacBio, Menlo Park, CA, USA), three genes (*mpclr4, mpdot1*, and *mplig4*) associated with genome stability were cloned and deduced as cDNA and amino acid sequence by SoftBerry’s FGENESH program (http://linuxl.softberry.com/berry.phtml). The phylogenetic tree was constructed by using MEGA10 software (Mega Limited, Auckland, New Zealand), and visualization analysis of results for multi-sequence alignment was performed by Genedoc software (PSC, Pittsburgh, USA) (Solovyev 2007).

### Construction of gene-inactivated transformants by ATMT

#### Strategy for vector construction in ATMT

Firstly, the deletion vectors were created following previous description (Zheng et al. 2022). For the deletion of gene *mpclr4,* a gene disruption cassette was firstly constructed, which consisted of upstream sequence, downstream sequence, and gene encoding hygromycin phosphotransferase (*hph*). Specifically, 750-bp upstream and 850-bp downstream homologous sequences of gene *mpclr4* were amplified with the primer pairs 5F/5R and 3F/3R, and a 2.2-kb *hph* expression fragment was amplified with primer pair *hph*F/*hph*R (shown in Supplemental Fig. S1a). All these amplicons were purified and ligated with *Kpn*I/*Xba*I digested pCAMBIA3300 in a ratio 1:1:1:2 by Gibson assembly according to the pEASY-Basic Seamless Cloning and Assembly Kit (TransGen, Beijing, China) to create vector pCCLR4. Analogous strategies were used to generate vectors pCDOT1 and pCLIG4 for deleting *mpdot1* and *mplig4*, respectively (shown in Supplemental Fig. S1a). Then, the created vectors were respectively introduced into *A. tumefaciens* EHA105 cells following previous procedure (Shao et al. 2009).

#### The procedure of ATMT

**Fig. 1:**
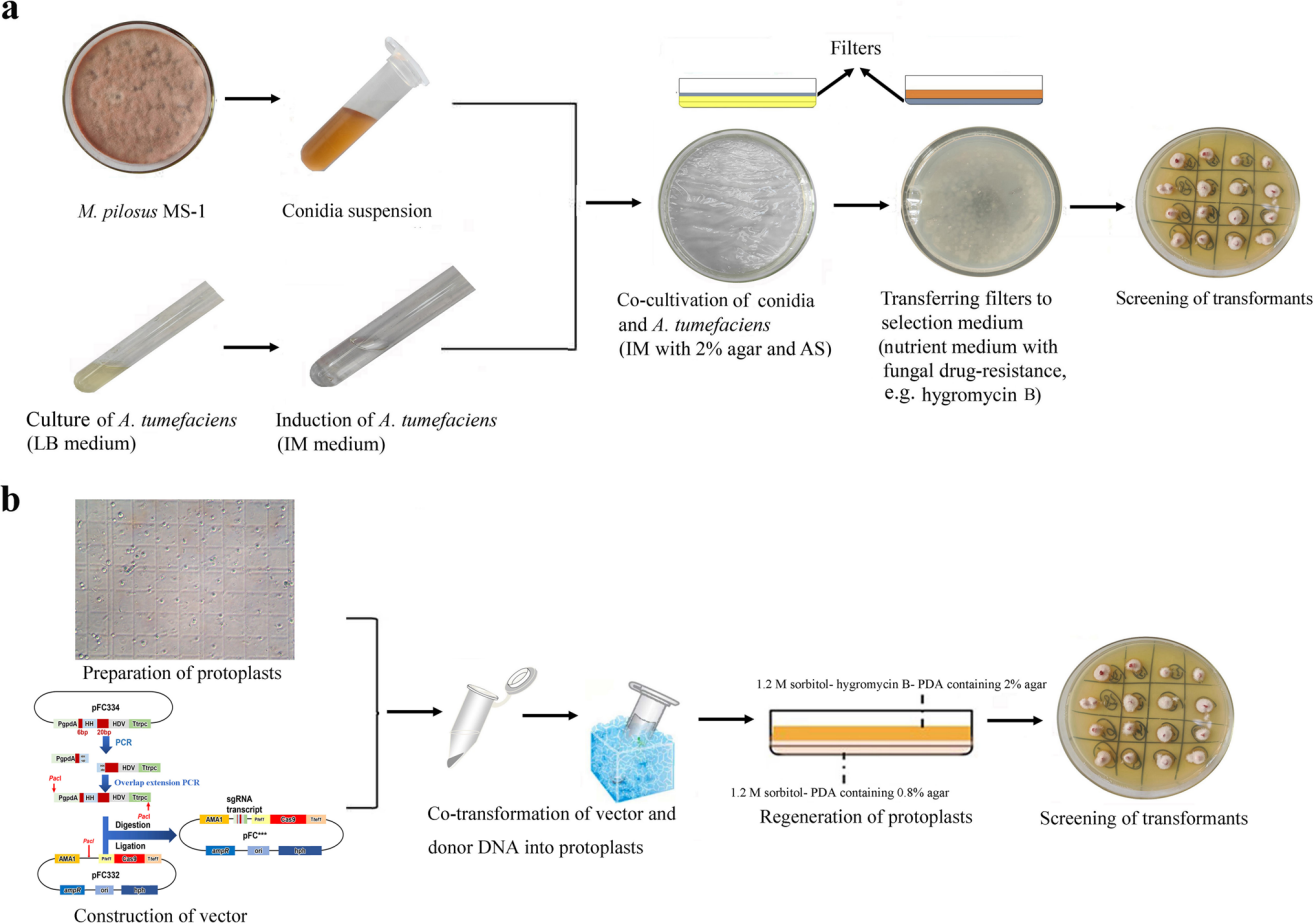
Demonstrations of gene manipulation methods used in *M. pilosus* MS-1. **a** The procedure of ATMT. **b** The procedure of CGES. HH is 5’-end hammerhead (HH), HDV is 3’-end hepatitis delta virus, and the protospacer consists of only 20 nucleotides that can recognize the target site by base pairing

The procedure of ATMT, as shown in Fig. 1a, included preparation of conidia suspension, induction of *A. tumefaciens*, co-cultivation of conidia and *A. tumefaciens*, and screening of transformants. Specifically, strain MS-1 was cultured at 28 °C on PDA for 10 days to prepare conidia, and the concentration of conidia suspension amounted to 4–6×10^5^/mL. Meanwhile, *A. tumefaciens* EHA105 cells carrying the deletion vector were cultured overnight at 28 °C in LB broth with 50 μg/mL kanamycin to an OD_600nm_ value of 0.8–1.2. Then, these *A. tumefaciens* EHA105 cells were collected and diluted with induction medium (IM, NaCl 0.3 g, Na_2_-EDTA·2H_2_O 1.3 mg, Na_2_MoO_4_·2H_2_O 0.5 mg, NH_4_NO_3_ 0.5 g, MgSO_4_·7H_2_O 0.6 g, ZnSO_4_·7H_2_O 0.5 mg, KH_2_PO_4_ 0.136 g, CaCl_2_·2H_2_O 0.01 g, CuSO_4_·5H_2_O 0.5 mg, H_3_BO_3_ 0.5 mg, FeSO_4_·7H_2_O 1 mg, glycerol 5 mL, distilled water to 1 L) containing 10 mM 2-(*N*-morpholino) ethanesulfonic acid (MES), 0.2 mM acetosyringone (AS), and 0.2% glucose (Glu) to an OD_600nm_ value of 0.5. After incubation for 6 h, *A. tumefaciens* EHA105 cells were mixed with the prepared conidia suspension of strain MS-1 in equal volume, and they were evenly spread on IM agar plates containing 10 mM MES, 0.5 mM AS, and 0.2% Glu covered with sterile cellophane (Solarbio, Beijing, China) for co-culture under dark condition at 28 °C. Following incubation for 2 days, the cellophane was transferred to a PDA plate containing 75 μg/mL hygromycin B and 100 μg/mL cefotaxime for growth at 28 °C. Those colonies grown on PDA containing 75 μg/mL hygromycin B were regarded as transformants.

### Construction of gene-inactivated transformants by CGES

#### Strategy for vector construction in CGES

The applicability of CGES in strain MS-1 was investigated as shown in Fig. 1b, including preparation of protoplasts, construction of vector containing expression cassette for Cas9 protein and sgRNA, co-transformation of vector and donor DNA into protoplasts, regeneration of protoplasts, and screening of transformants. For CGES, the sgRNA with a 20-bp protospacer was liberated from a larger transcript, which was ensured by employing the strong constitutive promoter *gpdA* (P*gpdA*) and the terminator *trpC* (T*trpC*) of *Aspergillus nidulans.* For vector construction, primer pairs of P*gdpA*-F/gRNA-*gene*-R and gRNA-*gene*-F/T*trpC*-R were used to amplify two DNA fragments containing a 6-bp reverse-complementing sequence and a 20-bp protospacer, which were inserted into the *Pac*I-digested pFC332 vector by Gibson assembly to generate pFCkoClr4, pFCkoDot1, and pFCkoLig4 (shown in Supplemental Fig. S1b). These reconstructed vectors were then confirmed by *Pst*I/*Asc*I digestion. The sequences of protospacer were filtered by the localized software sgRNAcas9_V3.0_GUI (Xie et al. 2014), and the online-based tools CHOPCHOP (http://chopchop.cbu.uib.no/) (Montague et al. 2014) and benchling (https://benchling.com) (Benchling, Boston, USA) for evaluating the targeting efficiency. For donor DNA, the upstream and downstream fragments of target site were amplified from genomic DNA of strain MS-1, and then these two amplicons were mixed in a 1:1 molar ratio by overlapping PCR.

#### The introduction of CGES into strain MS-1

To introduce this constructed CGES into strain MS-1, the protoplasts were prepared firstly. In total, 5 mL of conidia with a concentration of 10^6^/mL was inoculated into 100 mL YEME medium (0.3% yeast extract, 0.5% peptone, 0.3% malt extract, 1% glucose, 34% sucrose) and cultivated at 30 °C for 30 h with 220 rpm. Subsequently, the young germinated hyphae were filtered through four layers of lens tissue and washed twice with 10 mL of osmotic stabilizer (0.9 M NaCl, 5 mM DL-dithiothreitol, 10 mM Na_2_HPO_4_/NaH_2_PO_4_, pH 5.8). Following this, 200 mg of hyphae was transferred into 10 mL osmotic stabilizer, which contained 5 mg/mL of lysozyme (Guangdong Institute of Microbiology, Guangzhou, China), 15 mg/mL of snailase (Solarbio, Beijing, China), 5 mg/mL of lysing enzymes from *Arthrobacter luteus*, and 20 mg/mL of cellulase (Shanghai Yuanye Bio-Technology, Shanghai, China). After 3 h of hydrolysis at 30 °C with 80 rpm, the protoplasts were filtered by sterile syringe plugged with sterile cotton.

In the co-transformation stage, 200 μL of protoplasts (4–6×10^6^/mL) were mixed with at least 5 μg DNA (including vector and donor DNA) on ice for 40 min after washing twice by fresh STC buffer (1.2 M sorbitol, 10 mM Tris-HCl, 10 mM CaCl_2_, pH 7.5). Next, 1.25 mL of 50% PEG4000 solution (50% PEG 4000, 50 mM CaC1_2_, 50 mM Tris-HCl, pH 7.5) was mixed with the above suspension and incubated for 20 min at room temperature. For regeneration of protoplasts and screening of transformants, the aforementioned suspension was then cultured at 30 °C in double-layer plates consisting of semi-solid PDA and PDA with 0.5 mg/mL hygromycin B.

### Screening target gene-inactivated transformants

Firstly, the putative gene-inactivated transformants were subjected to PCR test. Specifically, the total genomic DNAs of these selected transformants were extracted with the CTAB (cetyl trimethyl ammonium bromide) method (Yang et al. 2014). PCR analysis was then performed using a specific primer pair (ORF-F/R), which was located on the open reading frame (ORF) region of the replacement site. Further, RT-qPCR was performed to verify the event of generating site specific gene inactivation. The RNA extraction was followed the protocol of TransZol Up Plus RNA Kit (TransGen Biotech, Beijing, China) for further cDNA synthesis. The RT-qPCR reaction system was as follows: 10 μL AceQ qPCR SYBR Green Master Mix, 0.4 μL of 2.5 μM forward primer, 0.4 μL of 2.5 μM reverse primer, 7.2 μL of ddH_2_O, and 2.0 μL of template cDNA. Thermal cycling conditions comprised 95 °C for 5 min, and 40 cycles consisting of 95 °C for 10 s and 60 °C for 30 s. *Actb* (beta-actin) served as the reference gene (Zhang et al. 2022), and relative expression levels were calculated by the 2^−ΔΔC^_T_ method according to the formula described previously (Livak and Schmittgen 2013).

### Calculation of GTE and GRF

In order to compare the applicability of ATMT and CGES as gene editing tools in *M. pilosus* MS-1, we compared the GTE and GRF between these methods. In this study, genetic transformation (GT) refers to the entry of exogenous genetic material into recipient, and a strain that can grow normally on PDA containing hygromycin B was considered as a transformant. The definition of gene replacement (GR) is that gene at specific locus is replaced by designed structure. Based on these two definitions, GTE and GRF are calculated as follows:

### Detection of colony morphology and the DNA damage repair ability

To evaluate the impact of genes *mpclr4*, *mpdot1*, and *mplig4* on colony growth, 1 μL conidia suspension (10^5^ conidia/mL) of the WT and gene-inactivated transformants was incubated on four typical media (PDA, MA, G25N, and CYA) for 7 days at 28 °C, and the colony morphologies were observed. The abilities of DDR for transformants were evaluated by analyzing the tolerances to DNA damaging agents. Specifically, 1 μL conidia suspension (10^4^/mL) was inoculated on PDA containing camptothecin (CPT, 2 μg/mL), methyl methanesulfonate (MMS, 0.1 mg/mL), hydroxyurea (HU, 3 mg/mL), and thiabendazole (TBZ, 0.02 μg/mL), respectively. The tolerances to DNA damaging agents are evaluated as follows:

$$\textrm{The}\ \textrm{tolerance}\ \left(\%\right)=\left[\frac{\textrm{Colony}\ \textrm{diameter}\ \left(\textrm{DNA}\ \textrm{damaging}\ \textrm{agent},\textrm{transformant}\right)}{\textrm{Colony}\ \textrm{diameter}\ \left(\textrm{PDA},\textrm{transformant}\right)}-\frac{\textrm{Colony}\ \textrm{diameter}\ \left(\textrm{DNA}\ \textrm{damaging}\ \textrm{agent},\textrm{WT}\right)}{\textrm{Colony}\ \textrm{diameter}\ \left(\textrm{PDA},\textrm{WT}\right)}\right]\times 100$$

To figure out the predominant DDR pathway in these transformants, the expression levels of key genes associated with NHEJ and homology directed repair (HDR) pathways (Mladenov and Iliakis 2011; Winczura et al. 2012) were assessed by RT-qPCR analysis as described above.

### Assessment of MK-producing capability

MK-producing fermentation process was performed as follows. Firstly, 200 μL conidia suspension (10^6^ conidia/mL) of the WT and mutant strains (Δ*mpclr4*, Δ*mpdot1*, and Δ*mplig4*) was inoculated into 10 mL primary culture media (glucose 50 g/L, peptone 10 g/L, NH_4_H_2_PO_4_ 2 g/L, MgSO_4_·7H_2_O 0.5 g/L, CaCl_2_ 0.1 g/L, potato juice instead of distilled water to 1 L, pH 6.0) and cultivated for 30 h at 30 °C with 180 rpm. Then, the pre-cultured inoculum was inoculated into 100 mL liquid-state fermentation medium (sucrose 30 g/L, soybean flour 38.75 g/L, MgSO_4_·7H_2_O 0.00105 mol/L, pH 5.5) at 30 °C for 3 days, followed by further incubation for 10 days at 24 °C. During the fermentation period, the fermentation broth was collected by filtering from the 5th day to the 13th day at 2-day interval, and then 1 mL obtained fermentation broth was freeze-dried to get residues. For extraction of MK, the obtained residues were re-dissolved in 1 mL 70% ethanol and extracted by ultrasound for 30 min, and then filtered through 0.22-μm membrane for HPLC analysis. The HPLC analysis was performed on a LC 20AT HPLC system (Shimadzu, Kyoto, Japan) equipped with SPD-M20A photodiode array detector and an Inertsil ODS-3 C18 column (4.6×250 mm; GL Sciences, Tokyo, Japan), and an isocratic mobile phase consisting of 0.05% phosphoric acid (pH 2.5) and acetonitrile (40: 60, v/v) run at a flow rate of 1 mL/min with detection wavelength of 238 nm.

#### Statistical analysis

All data were performed in triplicate, and they were presented as the mean ± standard deviation (SD). Significance was assessed by one-way analysis of variance (ANOVA) using GraphPad Prism 8.0 software (Boston, MA, USA).

All primers used in this study are listed in Supplemental Table S1 and S2.

## Results

### Sequence analysis of *mpclr4*, *mpdot1*, and *mplig4*

**Fig. 2:**
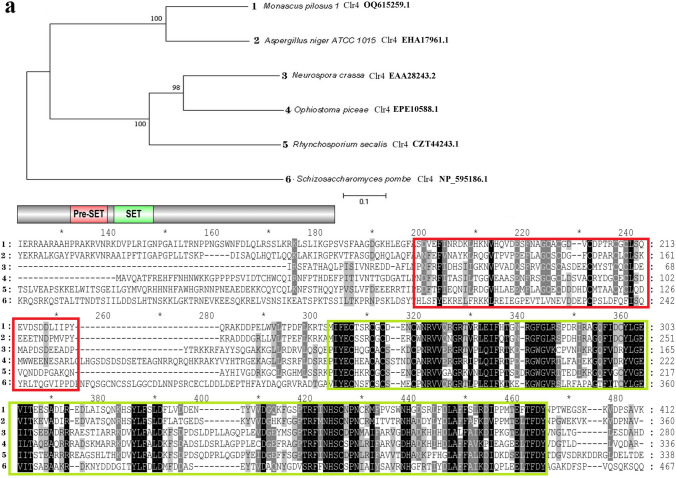

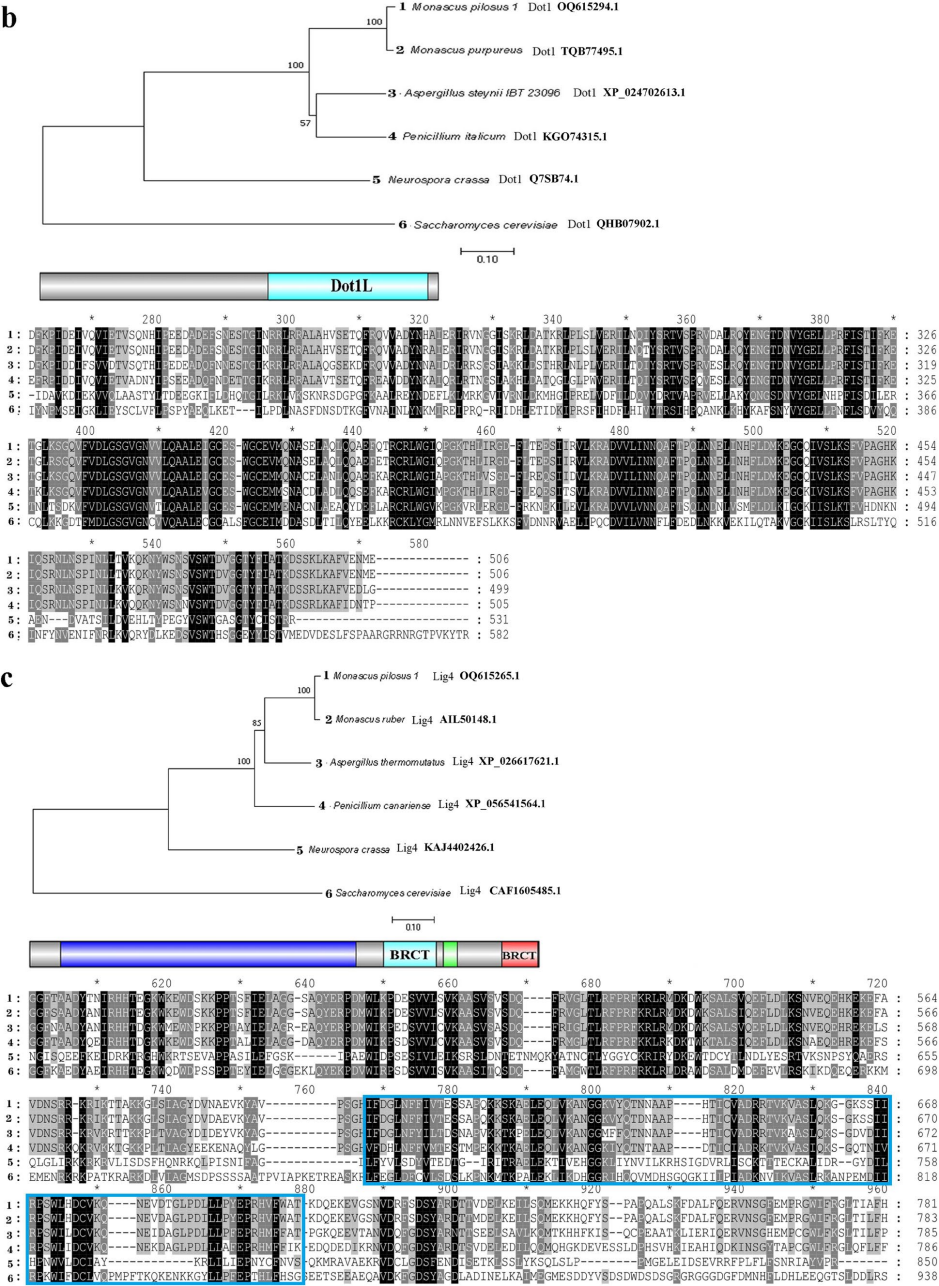
Sequence characteristic analysis. **a** Analysis of MpClr4. **b** Analysis of MpDot1. **c** Analysis of MpLig4. The branch length is inversely proportional to the homology between target sequences. The scale bar shows a length corresponding to 0.1 of the value

Based on the genome information of strain MS-1 sequenced by PacBio Sequel platform, three genes *mpclr4* (GenBank: OQ615259.1), *mpdot1* (GenBank: OQ615294.1), and *mplig4* (GenBank: OQ615265.1) were cloned and deduced as cDNA and the amino acid sequence by SoftBerry’s FGENESH program. They were deduced to encode 925 amino acids, 506 amino acids, and 890 amino acids, respectively. For further analysis, an online BLASTP was performed to search the homologs of these three proteins. As shown in Fig. 2, phylogenetic tree analysis suggested MpClr4, MpDot1, and MpLig4 were highly homologous to proteins Clr4, Dot1, and Lig4 in other species based on high similarity to amino acid sequences (*E* value < 10^-20^). Comparison of the domain architecture using Genedoc software revealed that Clr4 homologs possess the conserved SET (Su(var)3-9, Enhancer-of-zeste, Trithorax) domain (pfam00856), Dot1 homologs possess the conserved DOT1 (disruptor of telomeric silencing-1) domain (pfam08123), and Lig4 homologs possess the conserved BRCT DNA Ligase IV domain (cd17722).

### Comparison of GTE and GRF by the methods of ATMT and CGES

**Fig. 3:**
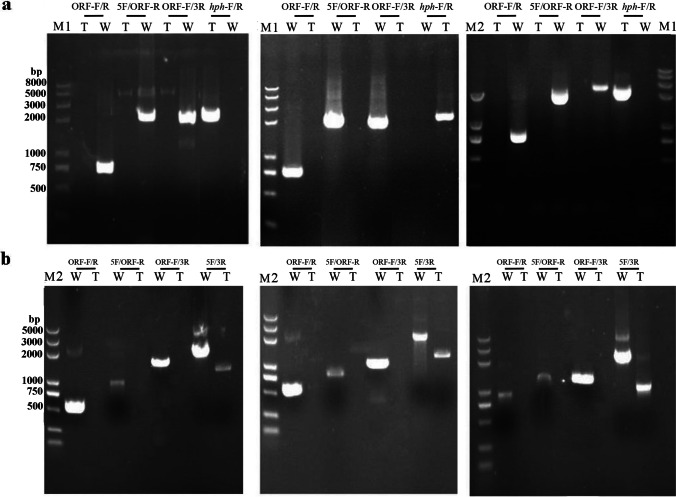
Nucleic acid electrophoresis of transformant verification for ATMT and CGES. **a** Verification of homologous recombination events for genes *mpclr4*, *mpdot1*, and *mplig4* in ATMT. **b** Verification of homologous recombination events for genes *mpclr4*, *mpdot1*, and *mplig4* in CGES. All “T” lanes mean fragment amplified by DNA extracted from transformant, all “W” lanes mean fragment amplified by DNA extracted from the WT, and all “M” lanes mean DNA markers

In this study, the number of conidia required for ATMT amounted to approximately 4×10^5^ conidia/mL, and 80–120 small colonies can re-grow on PDA containing hygromycin B. The PCR amplification using primer pair *hph* F/R suggested the deletion cassette has been integrated into the genome of those selected small colonies. Based on the calculation of GTE mentioned above, the GTE of ATMT ranged from 0.2 to 0.3‰. As presented in Fig. 3a, the DNAs of small colonies were used as a template for PCR detection, and no bands had been observed with the primers targeting ORF of specific gene, which was regarded as a site-specific gene replacement transformant. Additionally, the analysis of RT-qPCR could further confirm the generation of these gene-inactivated transformants by no detectably valid Ct values (data listed in Supplemental Table S3). For generating strain *∆mpclr4*, 197 transformants were obtained after two rounds of co-cultures, and only one strain had site-specific gene inactivation. One *∆mpdot1* strain was verified among 172 transformants through two rounds of co-cultures. Two *∆mplig4* strains were obtained from 307 transformants through three rounds of co-cultures. Thereby, the GRF were 5.1‰ for *∆mpclr4*, 7.3‰ for *∆mpdot1*, and 6.5‰ for *∆mplig4*, respectively.

By using CGES, a total of 60–80 transformants were obtained by PMT when approximately 4×10^6^ protoplasts were used for co-transformation. The strains grown on PDA containing hygromycin B were considered as transformants, so the GTE of CGES ranged from 0.015 to 0.02‰. As shown in Fig. 3b, a site-specific gene replacement transformant was obtained when no bands had been observed with the primers targeting ORF of specific gene. In CGES, two out of 73 transformants experienced site-specific gene editing event to *mpclr4*, three out of 80 transformants experienced site-specific gene editing event to *mpdot1*, and two out of 60 transformants experienced site-specific gene editing event to *mplig4*, respectively. Furthermore, RT-qPCR test confirmed the successful constructions of site-specific gene editing strains (data listed in Supplemental Table S3). Therefore, the GRF were 27.4‰ for *mpclr4*, 37.5‰ for *mpdot1*, and 33.3‰ for *mplig4* using CGES. In conclusion, the GTE of ATMT was about nine times higher than that of CGES, while the GRF of CGES was approximately five times than that of ATMT.

### Inactivation of these three genes has divergent effects on colony growth and tolerances to DNA damaging agents

**Fig. 4:**
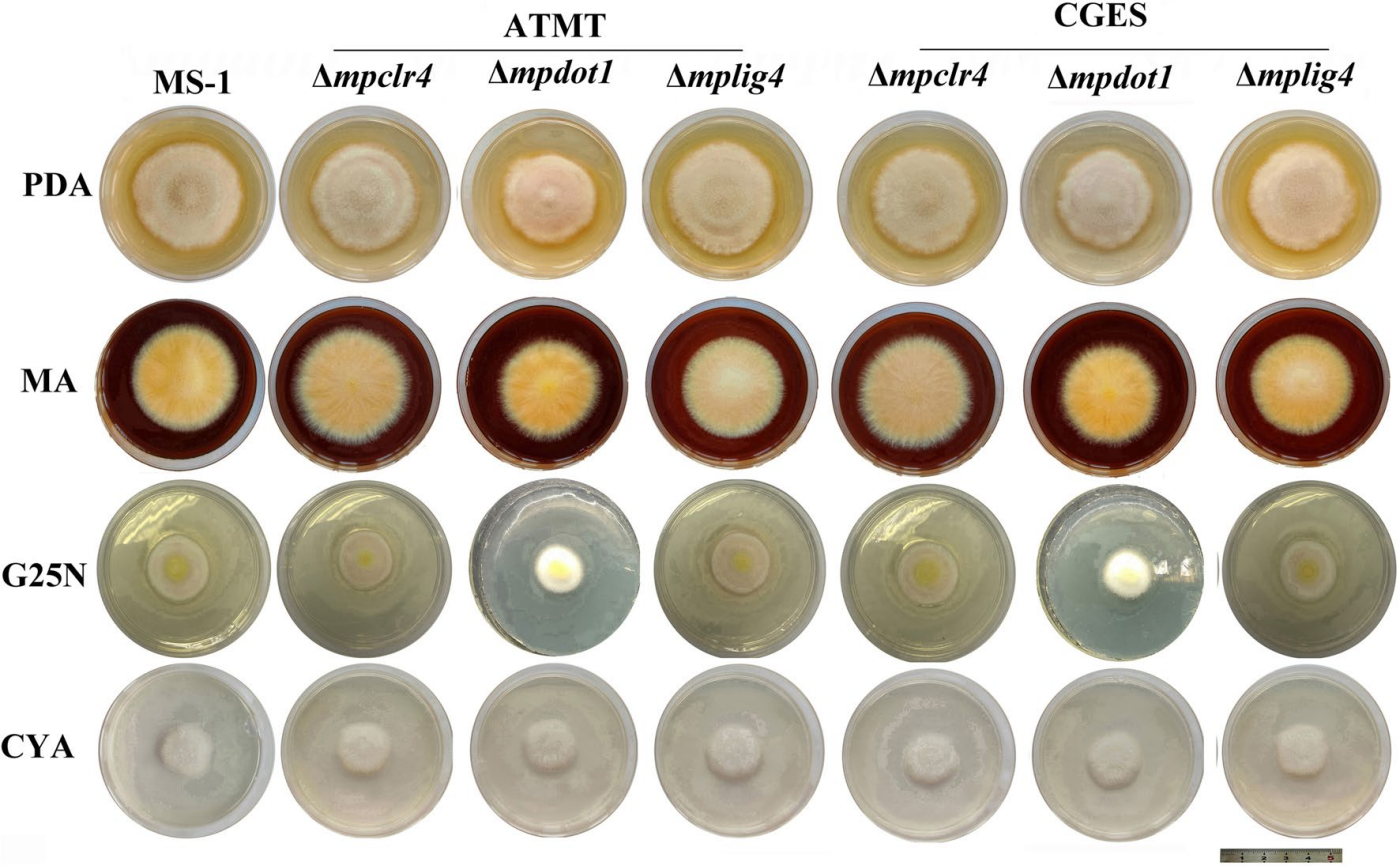
Colony morphology of the WT and transformants obtained by ATMT and CGES

**Fig. 5:**
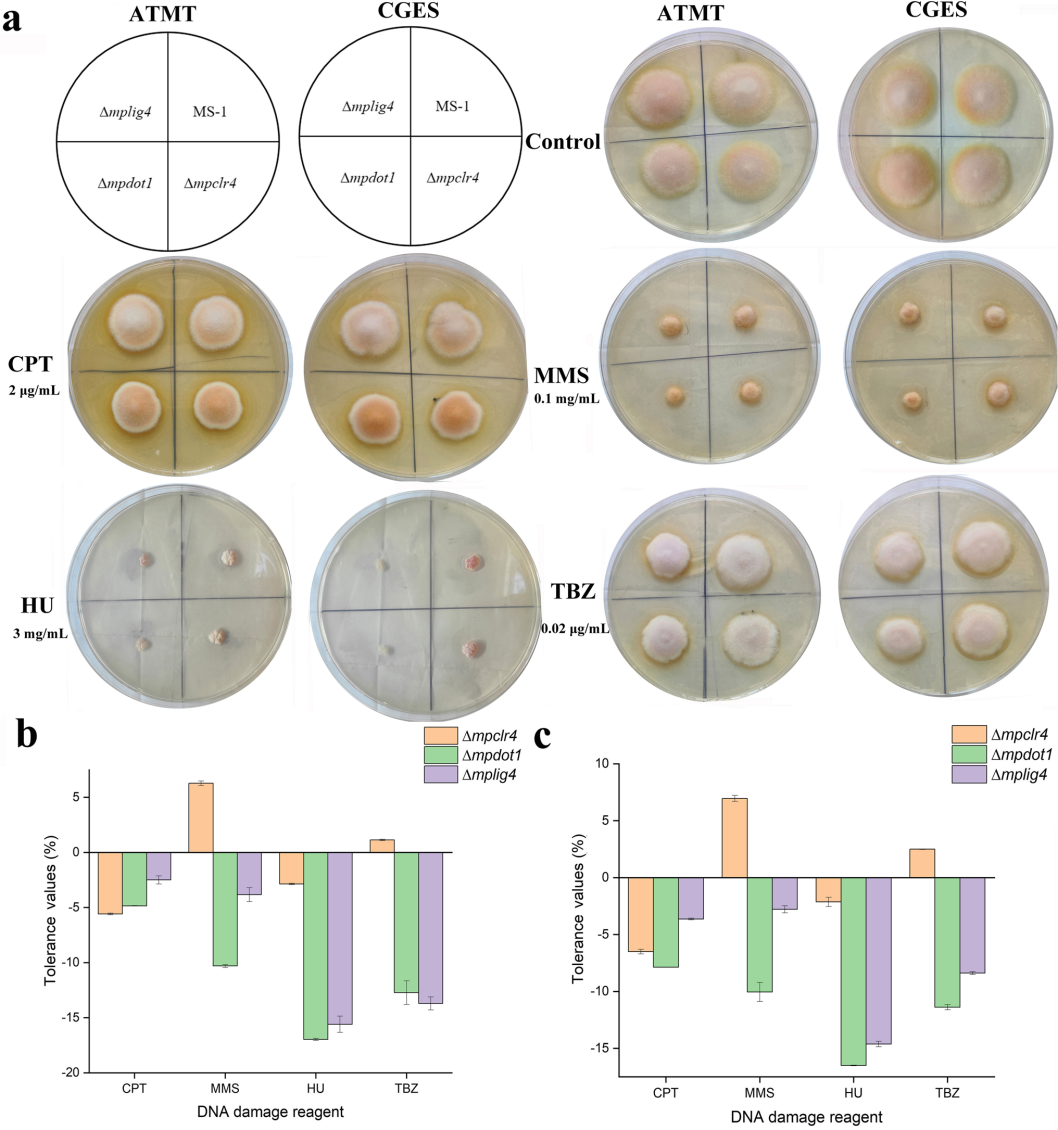
Tolerances to DNA damaging agents for the WT and transformants. **a** Colony morphology on PDA containing different DNA damaging agents for the WT and transformants obtained by ATMT and CGES. **b** Quantitative analysis of the tolerances to DNA damaging agents for transformants obtained by ATMT. **c** Quantitative analysis of the tolerances to DNA damaging agents for transformants obtained by CGES. The concentrations of CPT, MMS, HU, and TBZ are 2 μg/mL, 0.1 mg/mL, 3 mg/mL, and 0.02 μg/mL, respectively. A positive value represents an increased tolerance to DNA damaging agent, a negative value represents a decreased tolerance, and the magnitude of this value represents the strength of tolerance. Error bars represent SD

The WT and gene-inactivated transformants were inoculated on four typical media. As shown in Fig. 4, the visual observation showed that there was no obvious difference for transformants obtained by ATMT and CGES. The inactivation of these three genes did not cause severe growth defects, except that the colony diameter of strain Δ*mpdot1* was slightly smaller than the WT on PDA, MA, and G25N media. The tolerances of strains Δ*mpclr4,* Δ*mpdot1*, and Δ*mplig4* to DNA damaging agents are presented in Fig. 5. The transformants obtained by ATMT and CGES appeared similar trends in response to DNA damaging agents. The strain Δ*mpclr4* displayed increased tolerances to MMS and TBZ, but decreased tolerances to CPT and HU. Conversely, the strain Δ*mpdot1* showed decreased tolerances to four tested DNA damaging agents, and HU exerted the strongest inhibition to strain Δ*mpdot1* followed by TBZ, MMS, and CPT. Strain Δ*mplig4* exhibited the similar trends of tolerances to four tested DNA damaging agents with strain Δ*mpdot1.*

### Effects of three genes on the expression of the genes involved in DDR

**Fig. 6:**
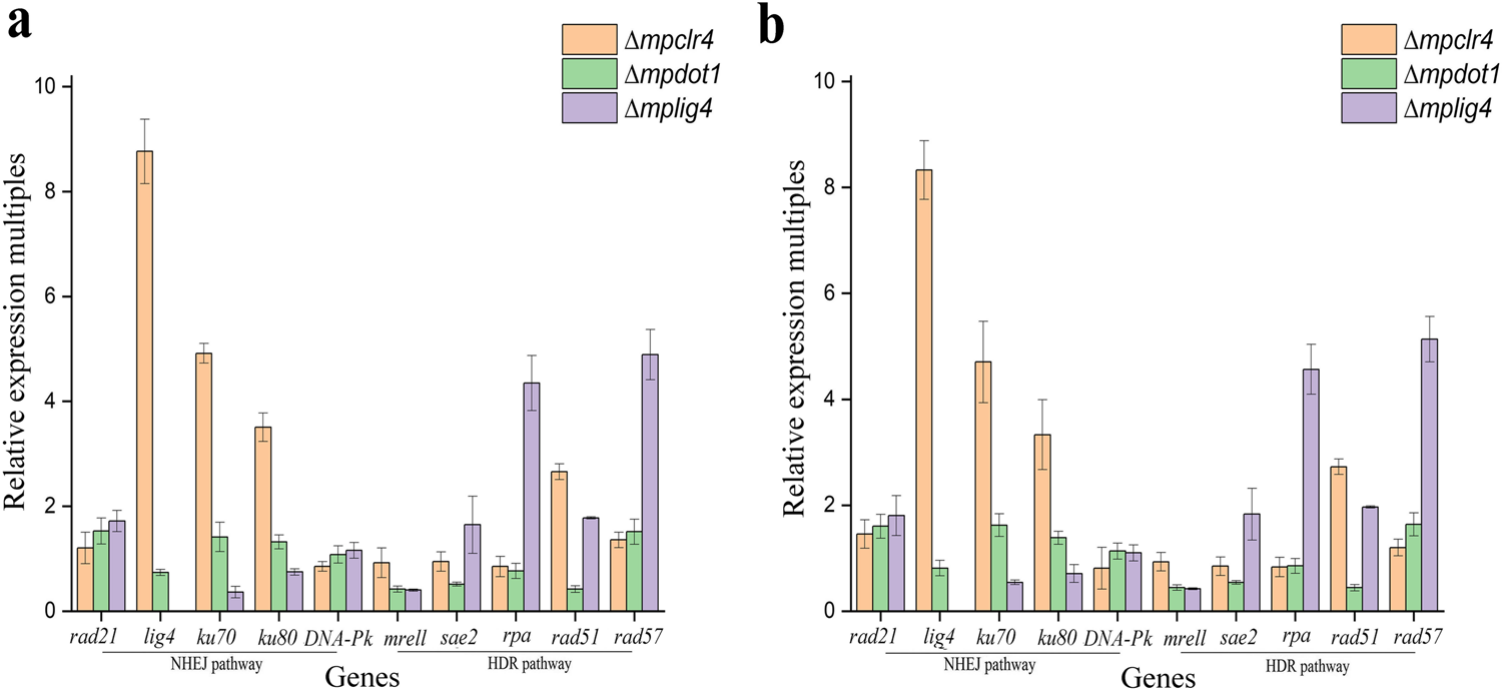
The expression level changes of genes related to DDR in transformants. **a** Expression level changes of genes related to DDR for transformants obtained by ATMT. **b** Expression level changes of genes related to DDR for transformants obtained by CGES. Error bars represent SD

Clr4 has been found to be a part of “Clr4 methyltransferase complex,” which comprises the cullin scaffold protein Cul4 to offer the binding site for DNA damage binding protein DDB1 (Jia et al. 2005). Published researches have demonstrated that the Dot1-mediated H3K79me was required for proper DDR in fungi (Liang et al. 2017; Wood et al. 2018). Thereby, the changes in expression levels of genes related to DDR were detected (Downs and Jackson 2004). Generally, Ku70, Ku80, Lig4, DNA-PK, and Rad21 participated in DDR via the NHEJ pathway, while Mrell, Sae2, Rad51, RPA, and Rad57 participated in DDR via the HDR pathway (Mladenov and Iliakis 2011; Winczura et al. 2012). As shown in Fig. 6, the effects on expression levels of genes related to DDR showed a consistent trend in transformants obtained by ATMT and CGES. In strain *∆mpclr4*, expression levels of most genes were increased, with the genes *lig4*, *ku70*, and *ku80* showing obvious activation. In strain *∆mpdot1*, the expression levels of genes *mrell*, *sae2*, and *rad51* were suppressed. The inactivation of *mplig4* inhibited the expression of genes related to the NHEJ pathway, while the expression levels of genes related to the HDR were enhanced, especially *rpa* and *rad57.*

### The inactivation of *mpclr4* led to the increased production of MK

**Fig. 7:**
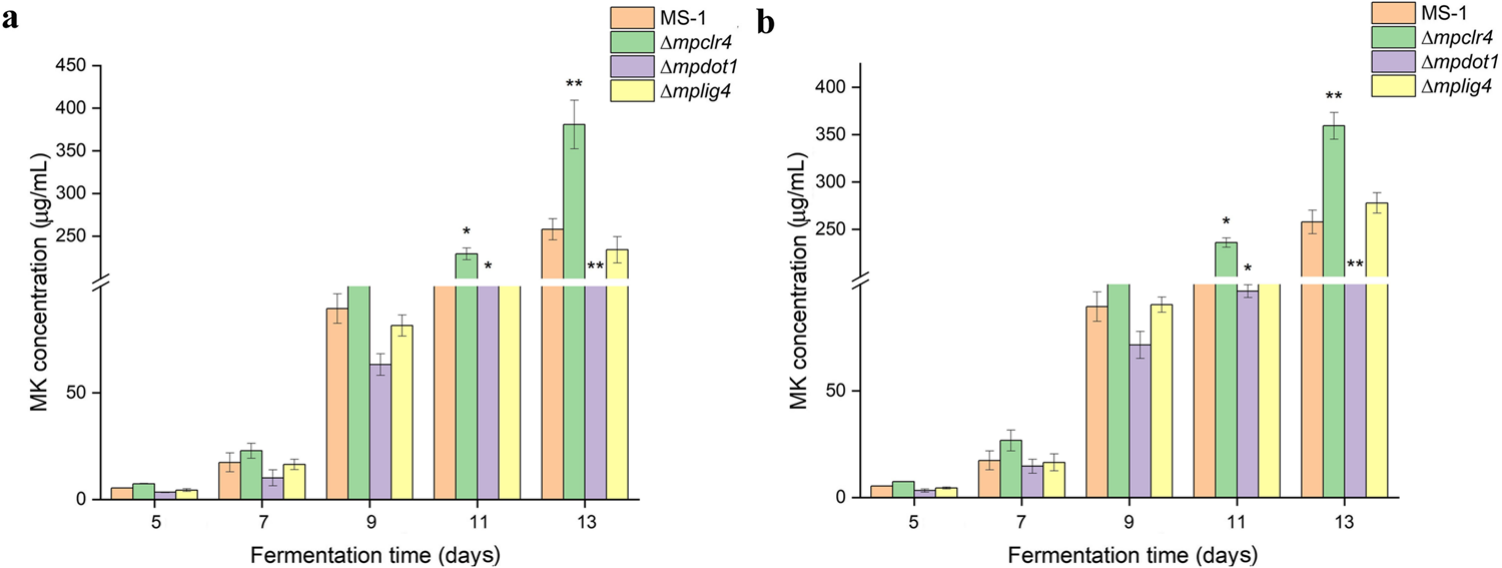
Yields of MK for the WT and transformants. **a** Yields of MK for the WT and transformants obtained by ATMT. **b** Yields of MK for the WT and transformants obtained by CGES. *p < 0.05, **p < 0.01; error bars represent SD

The strain *M. pilosus* MS-1 is an industrial strain used to produce MK (Shi et al. 2021) therefore, the production of MK was monitored during inactivation of *mpclr4*, *mpdot1*, and *mplig4*. As shown in Fig. 7, the transformants obtained by ATMT and CGES showed comparable fermentation capacity, and the contents of MK were increased with the fermentation time. The inactivation of *mpclr4* promoted the yield of MK, and the content of MK was increased by 52.6% until the end of fermentation. Conversely, the content of MK for strain *∆mpdot1* was significantly lower, yielding only 43.5% MK compared to that of the WT at the end of fermentation. The strain *∆mplig4* did not make a significant difference in producing MK compared to the WT. In order to obtain a strain suitable for commercial application, the strain Δ*mpclr4* obtained by CGES was then performed on plasmid removal through three passages without the addition of hygromycin B in PDA. By this operation, one *mpclr4*-inactivated strain that could not grow on PDA containing hygromycin B was obtained, which exhibited similar performance on producing MK with the *mpclr4*-inactivated strains with hygromycin B resistance, as shown in Supplemental Fig. S2.

## Discussion

Efficient genetic manipulation techniques are of great significance to explore gene function. As a molecular manipulation tool, ATMT has become the preferred method for filamentous fungi due to the simple process and flexible types of recipients (Bird and Bradshaw 1997; Lima et al. 2006)*.* For the application of ATMT in *Monascus* spp., it took 10–12 days to complete the process including construction of vector, induction of *A. tumefaciens*, co-cultivation of conidia and *A. tumefaciens*, and screening of transformants. Besides, the screening of transformants relies on the integration of a selectable marker (referred to *hph* in this study) into the recipient genome, which limits the commercial utilization of transformants. In addition, there are few genetic screening markers suitable for *Monascus* spp. Currently, only *hph* (Campoy et al. 2003) and *neo* (Xie et al. 2013) have been widely applied as genetic screening markers in *Monascus* spp. (Shao et al. 2014), further limiting the researches of multi-gene functions. In contrast, the CGES took 5–7 days including construction of vector, preparation of protoplasts, co-transformation of vector and donor DNA into protoplasts, regeneration of protoplasts, and screening of transformants. While the preparation of protoplasts is complex, the protocol of preparing protoplasts in this study has been proven to be suitable for various filamentous fungi (shown in Supplemental Fig. S3), which will provide a reference for other researchers. In this study, due to lacking expected visible phenotypic changes caused by these three genes, the screening of transformants obtained by CGES primarily relied on the expression of selectable marker *hph* in plasmid containing *AMA1* sequence, which is independently replicate without relying on chromosomes (Gems et al. 1991). Therefore, all small colonies growing on PDA with hygromycin B were firstly subjected to PCR detection, and those transformants without successful site-specific gene replacement were considered as off-targeted strains and eliminated. Then, RT-qPCR was used to further test the transcriptional inactivation of transformants that had undergone site-specific gene replacement confirmed by PCR. Through multiple passages on nutrient medium without resistance compounds hygromycin B, gene editing strains without screening marker could be successfully obtained, which would be favorable for commercial utilization and investigating the functions of multi-genes. For example, in *M. purpureus*, one citrinin-free transformant without integrating screening marker has been obtained by knocking out the gene responsible for citrinin biosynthesis and further applied to produce MPs (Liu et al. 2022). Ree et al. (2023) also applied CGES method to *M. ruber* for knocking out negative regulatory factors responsible for biosynthesizing MPs, and the yields of MPs were significantly enhanced. All these examples suggested that CGES is feasible for site-specific gene editing in industrial strains of *Monascus* species. Comparing the GRF between ATMT and CGES, the average GRF obtained by ATMT is 6.3‰, while the average GRF obtained by CGES is 32.7‰, indicating CGES is more suitable for gene knockout in *M. pilosus*.

During the fermentation process, *Monascus* spp. could be exposed to various kinds of environmental stresses, such as osmotic pressure, temperature, and ROS, which could induce various forms of DNA damages (Martha et al. 2015; Guan et al. 2017; Zeng et al. 2018). Normally, these DNA damages could be repaired by DDR, but the unrecoverable DDR of strains would cause mutation (Abril et al. 2010) and further lead to irreversible reduction of fermentation ability (Jena 2012). In yeast, the deletion of *clr4* promoted the activity of RNA-dependent RNA polymerases to amplify the aberrant transcripts resulting from DNA damage into small RNAs, which then activate DDR pathway (Hawley et al. 2017). Dot1 was considered to be required for proper DDR in fungi (Liang et al. 2017), and Lig4 was predicted to be crucial in the NHEJ (Pannunzio et al. 2018). Thereby, in order to explore the effect of these three proteins on DDR of *Monascus* spp., DNA damaging agents targeting different sites were added to media to simulate DNA damages. Specifically, the CPT inhibits topoisomerase activity (Sugimoto et al. 1990); MMS leads to DNA mismatches (Milo et al. 2019), HU may block DNA synthesis (Madaan et al. 2012); TBZ may inhibit the mitotic recombination of fungi (Tomlinson 2006). Although reduced tolerances to DNA damaging agents were observed in strains Δ*mpdot1* and Δ*mplig4*, the inactivation of *mpdot1* led to transcriptional inhibition of *rad51*, which functioned as a central rate-limiting protein of the HDR pathway and revealed the obstruction of the HDR pathway (Makino et al. 2020); the inactivation of *mplig4* led to the obvious defects in the NHEJ pathway. On the contrary, the inactivation of *mpclr4* increased the tolerance of strain Δ*mpclr4* to MMS and TBZ. Based on the analysis of RT-qPCR, the inactivation of *mpclr4* obviously promoted the expression levels of genes *ku70*, *ku80*, and *lig4*, which play central roles in NHEJ-mediated DDR (Downs and Jackson 2004). Thereby, these results indicated that these three encoded proteins had different functions on responding to various forms of DNA damages. In particular, the inactivation of *mpclr4* made strain MS-1 have better ability to repair DNA mismatches and mitotic recombination due to promoting the NHEJ pathway.

Filamentous fungi can produce a variety of SMs and are excellent producers of natural active substances (Keller 2019). MK is one of the important SMs produced by *Monascus* spp., which has been used to produce lipid-lowering drugs (Wen et al. 2020). Clr4 was the only “writer” of H3K9 methylation, which was considered a hallmark of gene repression and absolutely required for spreading heterochromatin regions (Corless et al. 2020; Zhang et al. 2008). The inactivation of *mpclr4* made chromatin looser, prompting the expansion of transcriptionally active regions (Corless et al. 2020), which stimulated MK synthesis, and ultimately increased the production of MK by 52.6%. Dot1-mediated H3K79 methylation was found to activate genes by blocking deacetylation of H4K16 by SIR proteins, which were related with heterochromatin formation (Zeng et al. 2018; Wood et al. 2018). The inactivation of *mpdot1* caused the diffusion of heterochromatin (Wood et al. 2018) and led to the reduced yield of MK. While MpLig4 plays an important role in DDR (Downs and Jackson 2004), it did not affect chromatin structure; thereby, strain Δ*mplig4* showed no difference in producing MK compared with the WT. In short, our study demonstrated that CGES was more efficient than ATMT for site-specific gene editing in the commercial strain *M. pilosus* MS-1 with high production of MK. More importantly, CGES has been proved to be a markerless, recyclable gene editing technique through plasmid removal, providing a feasible method for editing multiple genes in industrial fungi.

## Supplementary information

ESM 1 (PDF 675 kb)

## Data Availability

The data sets used during the present study are available from the corresponding author upon reasonable request.
