# Supplementary material for: CRISPR/Cas9 system is a suitable gene targeting editing tool to filamentous fungus Monascus pilosus
Source: Appl Microbiol Biotechnol. 2024 Jan 19;108:154. doi: 10.1007/s00253-023-12865-x (PMC10799099; doi:10.1007/s00253-023-12865-x)
Supplement: Supplementary file 1 [file 253_2023_12865_MOESM1_ESM.pdf]

Supplemental Material

Journal: Applied Microbiology and Biotechnology

Title: CRISPR/Cas9 system is a suitable gene targeting editing tool to filamentous fungus *Monascus pilosus*

The authors: Yunxia Gong[1], Shengfa Li[1], Qianrui Liu[1], Fusheng Chen[1,2], Yanchun Shao[1, 2, *]

The affiliations of the authors:

[1] College of Food Science and Technology, Huazhong Agricultural University, Wuhan, 430070, China.

[2] Hubei International Scientific and Technological Cooperation Base of Traditional Fermented Foods, Huazhong Agricultural University, Wuhan, 430070, China.

The e-mail address and telephone of the corresponding author: yanchunshao@mail.hzau.edu.cn, +86-27-87282111

Supplementary material legends

Table S1 Primers used for PCR in this study

Table S2 Primers used for RT-qPCR in this study

Table S3 Cycle threshold values of target genes detected by RT-qPCR

Fig. S1 Nucleic acid electrophoresis of vectors construction by Gibson assembly

Fig. S2 Yields of MK for strains MS-1, Δ*mpclr4* with plasmid and Δ*mpclr4* without plasmid

Fig. S3 Protoplasts released by different filamentous fungi

Table S1 Primers used for PCR in this study

Primers	Sequences 5'-3'	Amplified region
ATMT		
mpclr4-5F	CGGGCAGGACCGGACGGGGCGGTACCCACAGACAAGAAACACGTCAGCTA	730 bp- upstream homologous sequence for knockout of *mpclr4*
mpclr4-5R	CAATATCATCTTCTGTCGACCCACCAAACCTCAGGCATAG	
mpclr4-3F	GAGGTAATCCTTCTTTCTAGTTCACATTCGACTACAATCC	820 bp- downstream homologous sequence for knockout of *mpclr4*
mpclr4-3R	TTGCATGCCTGCAGGTCGACTCTAGACGCTTGGGAGACACG	
mpclr4-ORF-F	CCTCACCATCTCCCGCCTCA	ORF for *mpclr4*
mpclr4-ORF-F	TGCACCACGCGATTCCAG	
mpdot1-5F	GTACCCGGGGATCCTCTAGATTCAGAAGCGGAGCGCG	790 bp- upstream homologous sequence for knockout of *mpdot1*
mpdot1-5R	CAATATCATCTTCTGTCGACCCCTGCGCAGAGCAAATGAACTCGA	
mpdot1-3F	GAGGTAATCCTTCTTTCTAGGGGTCTATTTCCGTACTAT	890 bp- downstream homologous sequence for knockout of *mpdot1*
mpdot1-3R	ACGACGGCCAGTGCCAAGCTTGTTCAGCTAGTTAATTAATACT	
mpdot1-ORF-F	CCGAATAAGATGCGTCTTTCCTGGTT	ORF for *mpdot1*
mpdot1-ORF-R	AAGCAGACGGGGAAATACCGAC	
mplig4-5F	CGGGCAGGACCGGACGGGGCGGTACCTGGACCTAATGAAGGATAAAC	715 bp- upstream homologous sequence for knockout of *mplig4*
mplig4-5R	CAATATCATCTTCTGTCGACGGATGGCTGAACTGTCTCG	
mplig4-3F	GAGGTAATCCTTCTTTCTAGGATGAATCGGTTGTTCTTAGCG	860 bp- downstream homologous sequence for knockout of *mplig4*
mplig4-3R	CGACTCTAGAGGATCCCCGGGTCGTCCACGGTCGTATCTCG	
mplig4-ORF-F	CACGCTGAGAAACTCAACCCTCTC	ORF for *mplig4*
mplig4-ORF-R	ATCATCTTGTCGAACGAG	
hph-F	GTCGACAGAAGATGATATTG	Amplification of *hph* expression cassette
hph-R	CTAGAAAGAAGGATTACCTC	
CRISPR/Cas9 system		
P*gdpA*-F	CTGTTTCCGCTGAGGGTTTAATGCGTAAGCTCCCTAATTGGCCCATCCGGCA	Amplification of the transcript containing sgRNA
T*trpC*-R	CTGTCTCGGCTGAGGTCTTAATGAGCCAAGAGCGGATTCCTCAGTC	
gRNA-*mpclr4*-R	GAGCTTACTCGTTTCGTCCTCACGGACTCATCAGCGATGACGGTGATGTCTGC TCAAGC	Amplification of sgRNA containing 20 bp- protospacer for knockout of *mpclr4*
gRNA-*mpclr4*- F	GATGAGTCCGTGAGGACGAAACGAGTAAGCTCGTCCGATGATGAGAACACC TACGGTTTTAGAGCTAGAAATAG	
dDNA-*mpclr4*-5F	TCTATCTACCAAATACTACCCGACAGATGACA	540 bp- upstream donor DNA for knockout of *mpclr4*
dDNA-*mpclr4*-5R	TTTGGATAGTTTACGCTTGGGAGACACTGCACCACGCGATTCCAGCA	
dDNA-*mpclr4*-3F	GTGTCTCCCAAGCGTAAACTATCCAAA	510 bp- downstream donor DNA for knockout of *mpclr4*
dDNA-*mpclr4*-3R	CCAGGACGATGTCCAGTAAGAAGAGG	
mpclr4-ORF-F	AGCCATCTCGCAGAATAAGC	ORF for *mpclr4*
mpclr4-ORF-R	CGTAGTTGCACCAAAGACACCA	
gRNA-*mpdot1*-R	GAGCTTACTCGTTTCGTCCTCACGGACTCATCAGGAACGTCGGTGATGTCTGC TCAAGC	Amplification of sgRNA containing 20 bp- protospacer for knockout of *mpdot1*
gRNA-*mpdot1*-F	GATGAGTCCGTGAGGACGAAACGAGTAAGCTCGTCGAACGTATAAGGGTGA ATGGGTTTTAGAGCTAGAAATAG	
dDNA-*mpdot1*-5F	AACTGAGGATGATTCCCAGAAA	610 bp- upstream donor DNA for knockout of *mpdot1*
dDNA-*mpdot1*-5R	CGCTGACGATACGGATACCTCCCTTGATTAACAACCAGGCGTTT	
dDNA-*mpdot1*-3F	GGAGGTATCCGTATCGTCAGCG	520 bp- downstream donor DNA for knockout of *mpdot1*
dDNA-*mpdot1*-3R	ACCGCCCGAGTCGAGTCATC	
mpdot1-ORF-F	GAAGTTCGCCGTACACGTTGT	ORF for *mpdot1*
mpdot1-ORF-R	CAAGCGGGCTAGATTAAGTGATAG	
gRNA-*mplig4*-R	GAGCTTACTCGTTTCGTCCTCACGGACTCATCAGTCTGGACGGTGATGTCTGC TCAAGC	Amplification of sgRNA containing 20 bp- protospacer for knockout of *mplig4*
gRNA-*mplig4*-F	GATGAGTCCGTGAGGACGAAACGAGTAAGCTCGTCTCTGGATTTGAAATGCC CCGGTTTTAGAGCTAGAAATAG	
dDNA-*mplig4*-5F	CCAGATGAATCGGTTGTTCTTAGCG	520 bp- upstream donor DNA for knockout of *mplig4*
dDNA-*mplig4*-5R	TCTCCTCAATCCACTCCACCGTTACTCGTCCCTGTAGAAAGGAAACATTAGCC	
dDNA-*mplig4*-3F	AGTAACGGTGGAGTGGATTGAGGAGA	490 bp- downstream donor DNA for knockout of *mplig4*

dDNA-*mplig4*-3R	GGTACACGGAGGAAGCTAGGTGAGAA	
mplig4-ORF-F	ACTTTATAGGATCAATCGGTCACTCATTTCG	ORF for *mplig4*
mplig4-ORF-R	GCCGCCTGTCATTTCTTCATTTGTATT	

20

Table S2 Primers used for RT-qPCR in this study

Primers	Sequences 5’-3’	Note
β-actin F	TCTGGCACCACACATTCTACAA	For RT-qPCR test of gene *β-actin*
β-actin R	CGAAGACGATCTGGGTCATCT	
mpclr4 F	ACCTAGCCATCTCGCAGAATAAG	For RT-qPCR test of gene *mpclr4*
mpclr4 R	GACTATCGCCGTGGTTCCAT	
mpdot1 F	GCTCAGAAGCCTCAGATTCGTAA	For RT-qPCR test of gene *mpdot1*
mpdot1 R	TCGCTGCTCGGGAGTATGTC	
mplig4 F	CTTTACTGCCGCTGACTACACG	For RT-qPCR test of gene *mplig4*
mplig4 R	CTGACAGACACTGATGCTGCTTT	
rad21 F	ACGTGGATGTGGAAGCAGTGAA	For RT-qPCR test of gene *rad21*
rad21 R	GCCCAGTCGGTCCCGTAAGA	
ku70 F	AGAAATGGGAGATCCGCAAGG	For RT-qPCR test of gene *ku70*
ku70 R	TCCGAGGAACAAACCAGACGAG	
ku80 F	TATGGTGTTACTTGCACCTTCG	For RT-qPCR test of gene *ku80*
ku80 R	CAATGCGTTGATTTGTTCTCC	
dna-pk F	AAATCCTGCATCTTCGCCTGAG	For RT-qPCR test of gene *dnapk*
dna-pk R	GCGCCTGTGAGTGTCCTTCG	
mrell F	TCGAAGAAGCCAAGGCAGATT	For RT-qPCR test of gene *mrell*
mrell R	AATGATTGAGCAGCGAGGAACT	
sae2 F	CCAAGCGAGGCTGACAATACG	For RT-qPCR test of gene *sae2*
sae2 R	CCGCAGCAGTCCTCACTAACAC	
rpa F	CCTGTCTTTCTGATGGGTGC	For RT-qPCR test of gene *rpa*
rpa R	CATTAGATCGTTTGCGGTTGT	
rad51 F	GGCAAACCCACCTCGCTAA	For RT-qPCR test of gene *rad51*
rad51 R	GATCGCCAATTCCGTCTTCAT	
rad57 F	CCTGCCCTTGGCCTTGTTT	For RT-qPCR test of gene *rad57*
rad57 R	CCTTGTTCGGGTTTCAATGGTT	

Table S3 Cycle threshold values of target genes detected by RT-qPCR

Strain	Reference gene	Ct value	Strain	Target gene	Ct value
MS-1	*β-actin*	18.02	MS-1	*mpclr4*	20.21
MS-1	*β-actin*	18.14	MS-1	*mpclr4*	20.33
MS-1	*β-actin*	18.34	MS-1	*mpclr4*	20.34
Δ*mpclr4* (ATMT)	*β-actin*	19.02	Δ*mpclr4* (ATMT)	*mpclr4*	Not detected
Δ*mpclr4* (ATMT)	*β-actin*	18.89	Δ*mpclr4* (ATMT)	*mpclr4*	Not detected
Δ*mpclr4* (ATMT)	*β-actin*	19.21	Δ*mpclr4* (ATMT)	*mpclr4*	33.35
Δ*mpclr4* (CGES)	*β-actin*	17.48	Δ*mpclr4* (CGES)	*mpclr4*	Not detected
Δ*mpclr4* (CGES)	*β-actin*	17.84	Δ*mpclr4* (CGES)	*mpclr4*	Not detected
Δ*mpclr4* (CGES)	*β-actin*	17.88	Δ*mpclr4* (CGES)	*mpclr4*	33.67
MS-1	*β-actin*	18.02	MS-1	*mpdot1*	19.33
MS-1	*β-actin*	18.14	MS-1	*mpdot1*	19.45
MS-1	*β-actin*	18.34	MS-1	*mpdot1*	19.13
Δ*mpdot1* (ATMT)	*β-actin*	19.43	Δ*mpdot1* (ATMT)	*mpdot1*	33.21
Δ*mpdot1* (ATMT)	*β-actin*	20.24	Δ*mpdot1* (ATMT)	*mpdot1*	33.28
Δ*mpdot1* (ATMT)	*β-actin*	19.80	Δ*mpdot1* (ATMT)	*mpdot1*	Not detected
Δ*mpdot1* (CGES)	*β-actin*	17.27	Δ*mpdot1* (CGES)	*mpdot1*	Not detected
Δ*mpdot1* (CGES)	*β-actin*	17.26	Δ*mpdot1* (CGES)	*mpdot1*	33.45
Δ*mpdot1* (CGES)	*β-actin*	17.1	Δ*mpdot1* (CGES)	*mpdot1*	Not detected
MS-1	*β-actin*	18.02	MS-1	*mplig4*	19.73
MS-1	*β-actin*	18.14	MS-1	*mplig4*	19.33
MS-1	*β-actin*	18.34	MS-1	*mplig4*	19.45
Δ*mplig4* (ATMT)	*β-actin*	19.02	Δ*mplig4* (ATMT)	*mplig4*	Not detected
Δ*mplig4* (ATMT)	*β-actin*	18.95	Δ*mplig4* (ATMT)	*mplig4*	32.18
Δ*mplig4* (ATMT)	*β-actin*	19.55	Δ*mplig4* (ATMT)	*mplig4*	Not detected
Δ*mplig4* (CGES)	*β-actin*	17.18	Δ*mplig4* (CGES)	*mplig4*	33.93
Δ*mplig4* (CGES)	*β-actin*	17.28	Δ*mplig4* (CGES)	*mplig4*	Not detected
Δ*mplig4* (CGES)	*β-actin*	17.79	Δ*mplig4* (CGES)	*mplig4*	Not detected

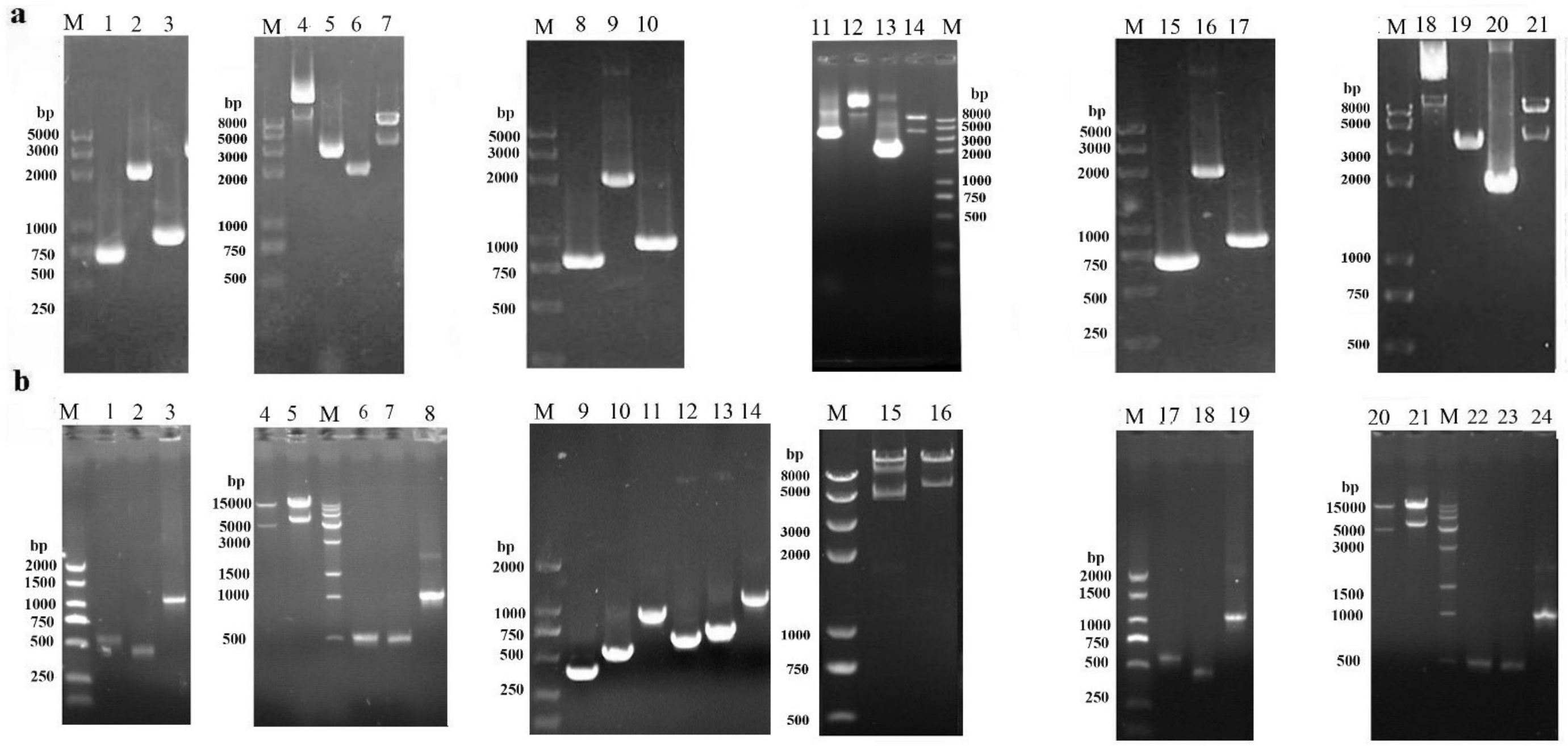

Fig. S1 Nucleic acid electrophoresis of vectors construction by Gibson assembly

a Construction of knockout vectors for target genes in ATMT. Lane 1/8/15: fragment amplified by primer pair gene-5F/5R (upstream for knockout of *mpclr4*, *mpdot1* and *mplig4*, respectively); lane 2/9/16: fragment amplified by primer pair *hph*-F/R using plasmid pSKH as template; lane 3/10/17: fragment amplified by primer pair gene-3F/3R (downstream for knockout of *mpclr4*, *mpdot1* and *mplig4*, respectively); lane 4/12/18: recombinant vector pCtarget extracted from *E. coli* DH5α; lane 5/11/19: knockout cassette amplified by primers gene-5F/3R using recombinant vector as template; lane 6/13/20: fragment amplified by primer pair *hph*-F/R using recombinant vector as template; lane 7/14/21: recombinant vector by double digestion; lane M: DNA marker. b Construction of knockout vectors for target genes in CGES. Lane 1/10/17: fragment amplified by primer pair P*gdpA*-F/gRNA-gene-R; lane 2/9/18: fragment amplified by primer pair gRNA-gene-F/T*trpC*-R; lane 3/11/19: sgRNA transcript amplified by primer pair P*gdpA*-F/T*trpC*-R; lane 4/15/20: vector pFC332 by double digestion; lane 5/16/21: recombinant vector pFCkotarget by double digestion; lane 6/13/22: fragment amplified by primer pair dDNA-gene-5F/5R (upstream donor DNA for knockout of *mpclr4*, *mpdot1* and *mplig4*, respectively); lane 7/12/23: fragment amplified by primer pair dDNA-gene-3F/3R (downstream donor DNA for knockout of *mpclr4*, *mpdot1* and *mplig4*, respectively); lane 8/14/24: donor DNA amplified by primer pair dDNA-gene-5F/3R for knockout of *mpclr4*, *mpdot1* and *mplig4*, respectively; lane M: DNA marker.

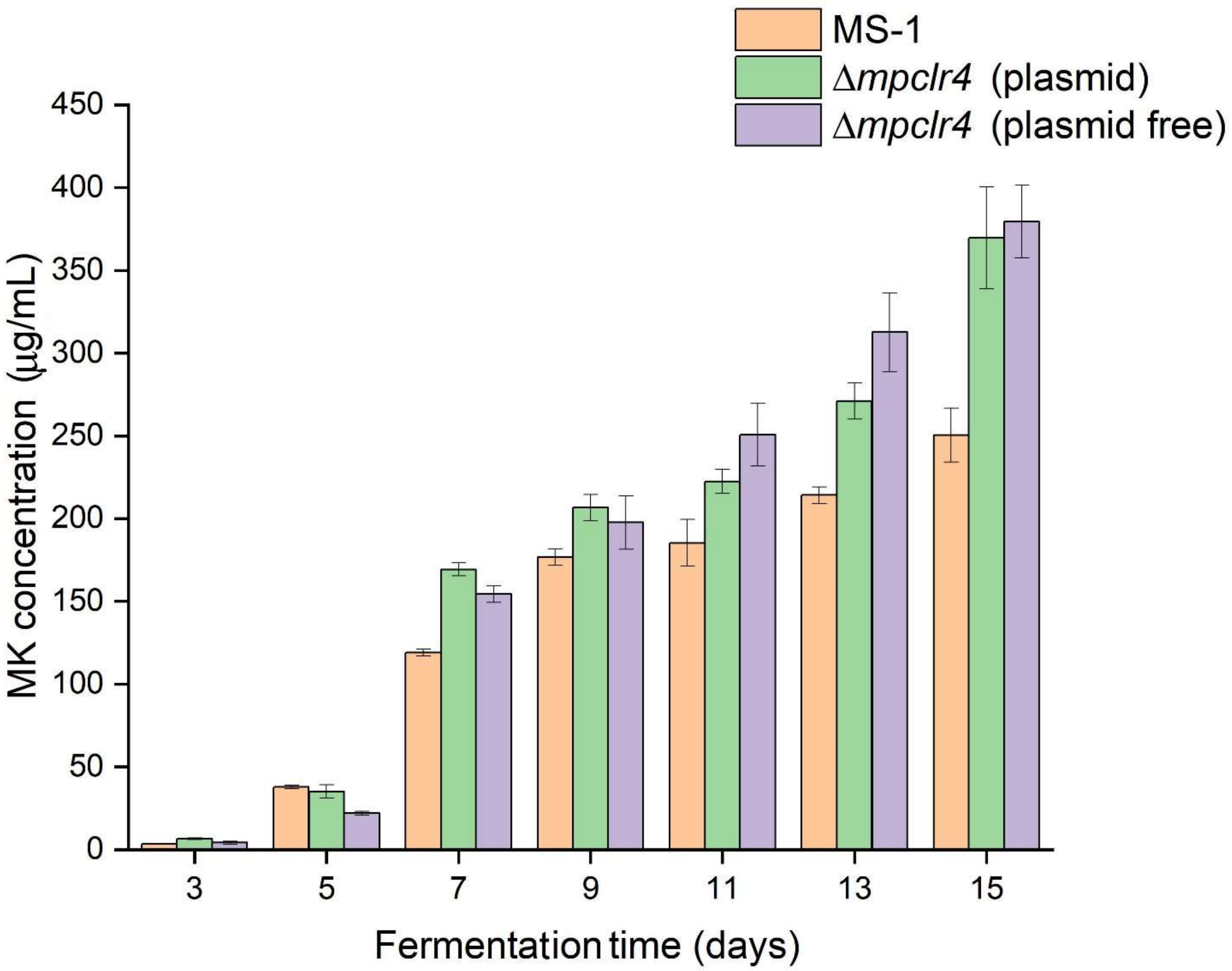

Fig. S2 Yields of MK for strains MS-1, Δ*mpclr4* with plasmid and Δ*mpclr4* without plasmid

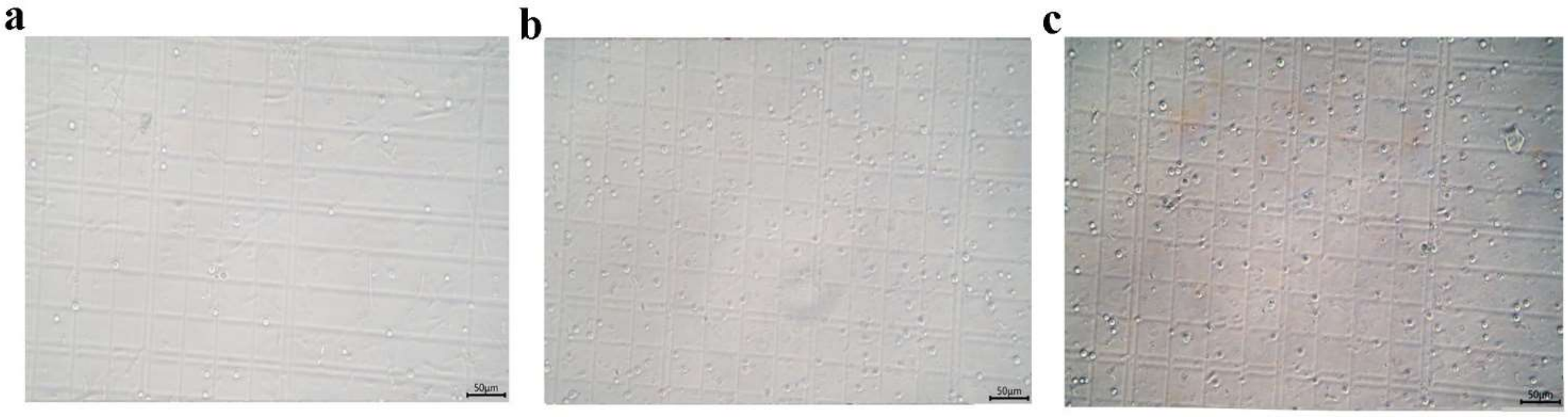

Fig. S3 Protoplasts released by different filamentous fungi

a Protoplasts of *Aspergillus oryzae* released by enzymatic hydrolysis. b Protoplasts of *Monascus pilosus* MS-1 released by enzymatic hydrolysis. c Protoplasts of *Monascus ruber* M7 released by enzymatic hydrolysis.
