# Supplementary material for: CRISPR/Cas9 system is a suitable gene targeting editing tool to filamentous fungus Monascus pilosus
Source: Appl Microbiol Biotechnol. 2024 Jan 19;108(1):154. doi: 10.1007/s00253-023-12865-x (PMC10799099; doi:10.1007/s00253-023-12865-x)
Supplement: Supplementary file 1 — (PDF 675 kb) [file 253_2023_12865_MOESM1_ESM.pdf]

Supplemental Material

Journal: Applied Microbiology and Biotechnology

Title: CRISPR/Cas9 system is a suitable gene targeting editing tool to filamentous fungus *Monascus pilosus*

The authors: Yunxia Gong[1], Shengfa Li[1], Qianrui Liu[1], Fusheng Chen[1,2], Yanchun Shao[1, 2, *]

The affiliations of the authors:

[1] College of Food Science and Technology, Huazhong Agricultural University, Wuhan, 430070, China.

[2] Hubei International Scientific and Technological Cooperation Base of Traditional Fermented Foods, Huazhong Agricultural University, Wuhan, 430070, China.

The e-mail address and telephone of the corresponding author: yanchunshao@mail.hzau.edu.cn, +86-27-87282111

## Supplementary material legends

**Table S1** Primers used for PCR in this study

**Table S2** Primers used for RT-qPCR in this study

**Table S3** Cycle threshold values of target genes detected by RT-qPCR

**Fig. S1** Nucleic acid electrophoresis of vectors construction by Gibson assembly

**Fig. S2** Yields of MK for strains MS-1, Δ*mpclr4* with plasmid and Δ*mpclr4* without plasmid

**Fig. S3** Protoplasts released by different filamentous fungi

Table S1 Primers used for PCR in this study

| Primers | Sequences 5’-3’ | Amplified region |
|---|---|---|
| ATMT | | |
| *mpclr4*-5F | CGGGCAGGACCGGACGGGGCGGTACCCACAGACAAGAAACACGTCAGCTA | 730 bp- upstream homologous sequence for knockout of *mpclr4* |
| *mpclr4*-5R | CAATATCATCTTCTGTCGACCCACCAAACCTCAGGCATAG | |
| *mpclr4*-3F | GAGGTAATCCTTCTTTCTAGTTCACATTCGACTACAATCC | 820 bp- downstream homologous sequence for knockout of *mpclr4* |
| *mpclr4*-3R | TTGCATGCCTGCAGGTCGACTCTAGACGCTTGGGAGACACG | |
| *mpclr4*-ORF-F | CCTCACCATCTCCCGCCTCA | ORF for *mpclr4* |
| *mpclr4*-ORF-F | TGCACCACGCGATTCCAG | |
| *mpdot1*-5F | GTACCCGGGGATCCTCTAGATTCAGAAGCGGAGCGCG | 790 bp- upstream homologous sequence for knockout of *mpdot1* |
| *mpdot1*-5R | CAATATCATCTTCTGTCGACCCCTGCGCAGAGCAAATGAACTCGA | |
| *mpdot1*-3F | GAGGTAATCCTTCTTTCTAGGGGTCTATTTCCGTACTAT | 890 bp- downstream homologous sequence for knockout of *mpdot1* |
| *mpdot1*-3R | ACGACGGCCAGTGCCAAGCTTGTTCAGCTAGTTAATTAATACT | |
| *mpdot1*-ORF-F | CCGAATAAGATGCGTCTTTCCTGGTT | ORF for *mpdot1* |
| *mpdot1*-ORF-R | AAGCAGACGGGGAAATACCGAC | |
| *mplig4*-5F | CGGGCAGGACCGGACGGGGCGGTACCTGGACCTAATGAAGGATAAAC | 715 bp- upstream homologous sequence for knockout of *mplig4* |
| *mplig4*-5R | CAATATCATCTTCTGTCGACGGATGGCTGAACTGTCTCG | |
| *mplig4*-3F | GAGGTAATCCTTCTTTCTAGGATGAATCGGTTGTTCTTAGCG | 860 bp- downstream homologous sequence for knockout of *mplig4* |
| *mplig4*-3R | CGACTCTAGAGGATCCCCGGGTCGTCCACGGTCGTATCTCG | |
| *mplig4*-ORF-F | CACGCTGAGAAACTCAACCCTCTC | ORF for *mplig4* |
| *mplig4*-ORF-R | ATCATCTTGTCGAACGAG | |
| *hph*-F | GTCGACAGAAGATGATATTG | Amplification of *hph* expression cassette |
| *hph*-R | CTAGAAAGAAGGATTACCTC | |
| CRISPR/Cas9 system | | |
| P*gdpA*-F | CTGTTTCCGCTGAGGGTTTAATGCGTAAGCTCCCTAATTGGCCCATCCGGCA | Amplification of the transcript containing sgRNA |
| T*trpC*-R | CTGTCTCGGCTGAGGTCTTAATGAGCCAAGAGCGGATTCCTCAGTC | |
| gRNA-*mpclr4*-R | GAGCTTACTCGTTTCGTCCTCACGGACTCATCAGCGATGACGGTGATGTCTGC TCAAGC | Amplification of sgRNA containing 20 bp- protospacer for knockout of *mpclr4* |
| gRNA-*mpclr4*- F | GATGAGTCCGTGAGGACGAAACGAGTAAGCTCGTCCGATGATGAGAACACC TACGGTTTTAGAGCTAGAAATAG | |
| dDNA-*mpclr4*-5F | TCTATCTACCAAATACTACCCGACAGATGACA | 540 bp- upstream donor DNA for knockout of *mpclr4* |
| dDNA-*mpclr4*-5R | TTTGGATAGTTTACGCTTGGGAGACACTGCACCACGCGATTCCAGCA | |
| dDNA-*mpclr4*-3F | GTGTCTCCCAAGCGTAAACTATCCAAA | 510 bp- downstream donor DNA for knockout of *mpclr4* |
| dDNA-*mpclr4*-3R | CCAGGACGATGTCCAGTAAGAAGAGG | |
| *mpclr4*-ORF-F | AGCCATCTCGCAGAATAAGC | ORF for *mpclr4* |
| *mpclr4*-ORF-R | CGTAGTTGCACCAAAGACACCA | |
| gRNA-*mpdot1*-R | GAGCTTACTCGTTTCGTCCTCACGGACTCATCAGGAACGTCGGTGATGTCTGC TCAAGC | Amplification of sgRNA containing 20 bp- protospacer for knockout of *mpdot1* |
| gRNA-*mpdot1*-F | GATGAGTCCGTGAGGACGAAACGAGTAAGCTCGTCGAACGTATAAGGGTGA ATGGGTTTTAGAGCTAGAAATAG | |
| dDNA-*mpdot1*-5F | AACTGAGGATGATTCCCAGAAA | 610 bp- upstream donor DNA for knockout of *mpdot1* |
| dDNA-*mpdot1*-5R | CGCTGACGATACGGATACCTCCCTTGATTAACAACCAGGCGTTT | |
| dDNA-*mpdot1*-3F | GGAGGTATCCGTATCGTCAGCG | 520 bp- downstream donor DNA for knockout of *mpdot1* |
| dDNA-*mpdot1*-3R | ACCGCCCGAGTCGAGTCATC | |
| *mpdot1*-ORF-F | GAAGTTCGCCGTACACGTTGT | ORF for *mpdot1* |
| *mpdot1*-ORF-R | CAAGCGGGCTAGATTAAGTGATAG | |
| gRNA-*mplig4*-R | GAGCTTACTCGTTTCGTCCTCACGGACTCATCAGTCTGGACGGTGATGTCTGC TCAAGC | Amplification of sgRNA containing 20 bp- protospacer for knockout of *mplig4* |
| gRNA-*mplig4*-F | GATGAGTCCGTGAGGACGAAACGAGTAAGCTCGTCTCTGGATTTGAAATGCC CCGGTTTTAGAGCTAGAAATAG | |
| dDNA-*mplig4*-5F | CCAGATGAATCGGTTGTTCTTAGCG | 520 bp- upstream donor DNA for knockout of *mplig4* |
| dDNA-*mplig4*-5R | TCTCCTCAATCCACTCCACCGTTACTCGTCCCTGTAGAAAGGAAACATTAGCC | |
| dDNA-*mplig4*-3F | AGTAACGGTGGAGTGGATTGAGGAGA | 490 bp- downstream donor DNA for knockout of *mplig4* |

| | | |
|---|---|---|
| dDNA-*mplig4*-3R | GGTACACGGAGGAAGCTAGGTGAGAA | |
| *mplig4*-ORF-F | ACTTTATAGGATCAATCGGTCACTCATTTCG | ORF for *mplig4* |
| *mplig4*-ORF-R | GCCGCCTGTCATTTCTTCATTTGTATT | |

Table S2 Primers used for RT-qPCR in this study

| Primers | Sequences 5’-3’ | Note |
|---|---|---|
| *β-actin* F | TCTGGCACCACACATTCTACAA | For RT-qPCR test of gene *β-actin* |
| *β-actin* R | CGAAGACGATCTGGGTCATCT | |
| *mpclr4* F | ACCTAGCCATCTCGCAGAATAAG | For RT-qPCR test of gene *mpclr4* |
| *mpclr4* R | GACTATCGCCGTGGTTCCAT | |
| *mpdot1* F | GCTCAGAAGCCTCAGATTCGTAA | For RT-qPCR test of gene *mpdot1* |
| *mpdot1* R | TCGCTGCTCGGGAGTATGTC | |
| *mplig4* F | CTTTACTGCCGCTGACTACACG | For RT-qPCR test of gene *mplig4* |
| *mplig4* R | CTGACAGACACTGATGCTGCTTT | |
| *rad21* F | ACGTGGATGTGGAAGCAGTGAA | For RT-qPCR test of gene *rad21* |
| *rad21* R | GCCCAGTCGGTCCCGTAAGA | |
| *ku70* F | AGAAATGGGAGATCCGCAAGG | For RT-qPCR test of gene *ku70* |
| *ku70* R | TCCGAGGAACAAACCAGACGAG | |
| *ku80* F | TATGGTGTTACTTGCACCTTCG | For RT-qPCR test of gene *ku80* |
| *ku80* R | CAATGCGTTGATTTGTTCTCC | |
| *dna-pk* F | AAATCCTGCATCTTCGCCTGAG | For RT-qPCR test of gene *dnapk* |
| *dna-pk* R | GCGCCTGTGAGTGTCCTTCG | |
| *mrell* F | TCGAAGAAGCCAAGGCAGATT | For RT-qPCR test of gene *mrell* |
| *mrell* R | AATGATTGAGCAGCGAGGAACT | |
| *sae2* F | CCAAGCGAGGCTGACAATACG | For RT-qPCR test of gene *sae2* |
| *sae2* R | CCGCAGCAGTCCTCACTAACAC | |
| *rpa* F | CCTGTCTTTCTGATGGGTGC | For RT-qPCR test of gene *rpa* |
| *rpa* R | CATTAGATCGTTTGCGGTTGT | |
| *rad51* F | GGCAAACCCACCTCGCTAA | For RT-qPCR test of gene *rad51* |
| *rad51* R | GATCGCCAATTCCGTCTTCAT | |
| *rad57* F | CCTGCCCTTGGCCTTGTTT | For RT-qPCR test of gene *rad57* |
| *rad57* R | CCTTGTTCGGGTTTCAATGGTT | |

Table S3 Cycle threshold values of target genes detected by RT-qPCR

| Strain | Reference gene | Ct value | Strain | Target gene | Ct value |
|---|---|---|---|---|---|
| MS-1 | *β-actin* | 18.02 | MS-1 | *mpclr4* | 20.21 |
| MS-1 | *β-actin* | 18.14 | MS-1 | *mpclr4* | 20.33 |
| MS-1 | *β-actin* | 18.34 | MS-1 | *mpclr4* | 20.34 |
| Δ*mpclr4* (ATMT) | *β-actin* | 19.02 | Δ*mpclr4* (ATMT) | *mpclr4* | Not detected |
| Δ*mpclr4* (ATMT) | *β-actin* | 18.89 | Δ*mpclr4* (ATMT) | *mpclr4* | Not detected |
| Δ*mpclr4* (ATMT) | *β-actin* | 19.21 | Δ*mpclr4* (ATMT) | *mpclr4* | 33.35 |
| Δ*mpclr4* (CGES) | *β-actin* | 17.48 | Δ*mpclr4* (CGES) | *mpclr4* | Not detected |
| Δ*mpclr4* (CGES) | *β-actin* | 17.84 | Δ*mpclr4* (CGES) | *mpclr4* | Not detected |
| Δ*mpclr4* (CGES) | *β-actin* | 17.88 | Δ*mpclr4* (CGES) | *mpclr4* | 33.67 |
| MS-1 | *β-actin* | 18.02 | MS-1 | *mpdot1* | 19.33 |
| MS-1 | *β-actin* | 18.14 | MS-1 | *mpdot1* | 19.45 |
| MS-1 | *β-actin* | 18.34 | MS-1 | *mpdot1* | 19.13 |
| Δ*mpdot1* (ATMT) | *β-actin* | 19.43 | Δ*mpdot1* (ATMT) | *mpdot1* | 33.21 |
| Δ*mpdot1* (ATMT) | *β-actin* | 20.24 | Δ*mpdot1* (ATMT) | *mpdot1* | 33.28 |
| Δ*mpdot1* (ATMT) | *β-actin* | 19.80 | Δ*mpdot1* (ATMT) | *mpdot1* | Not detected |
| Δ*mpdot1* (CGES) | *β-actin* | 17.27 | Δ*mpdot1* (CGES) | *mpdot1* | Not detected |
| Δ*mpdot1* (CGES) | *β-actin* | 17.26 | Δ*mpdot1* (CGES) | *mpdot1* | 33.45 |
| Δ*mpdot1* (CGES) | *β-actin* | 17.1 | Δ*mpdot1* (CGES) | *mpdot1* | Not detected |
| MS-1 | *β-actin* | 18.02 | MS-1 | *mplig4* | 19.73 |
| MS-1 | *β-actin* | 18.14 | MS-1 | *mplig4* | 19.33 |
| MS-1 | *β-actin* | 18.34 | MS-1 | *mplig4* | 19.45 |
| Δ*mplig4* (ATMT) | *β-actin* | 19.02 | Δ*mplig4* (ATMT) | *mplig4* | Not detected |
| Δ*mplig4* (ATMT) | *β-actin* | 18.95 | Δ*mplig4* (ATMT) | *mplig4* | 32.18 |
| Δ*mplig4* (ATMT) | *β-actin* | 19.55 | Δ*mplig4* (ATMT) | *mplig4* | Not detected |
| Δ*mplig4* (CGES) | *β-actin* | 17.18 | Δ*mplig4* (CGES) | *mplig4* | 33.93 |
| Δ*mplig4* (CGES) | *β-actin* | 17.28 | Δ*mplig4* (CGES) | *mplig4* | Not detected |
| Δ*mplig4* (CGES) | *β-actin* | 17.79 | Δ*mplig4* (CGES) | *mplig4* | Not detected |

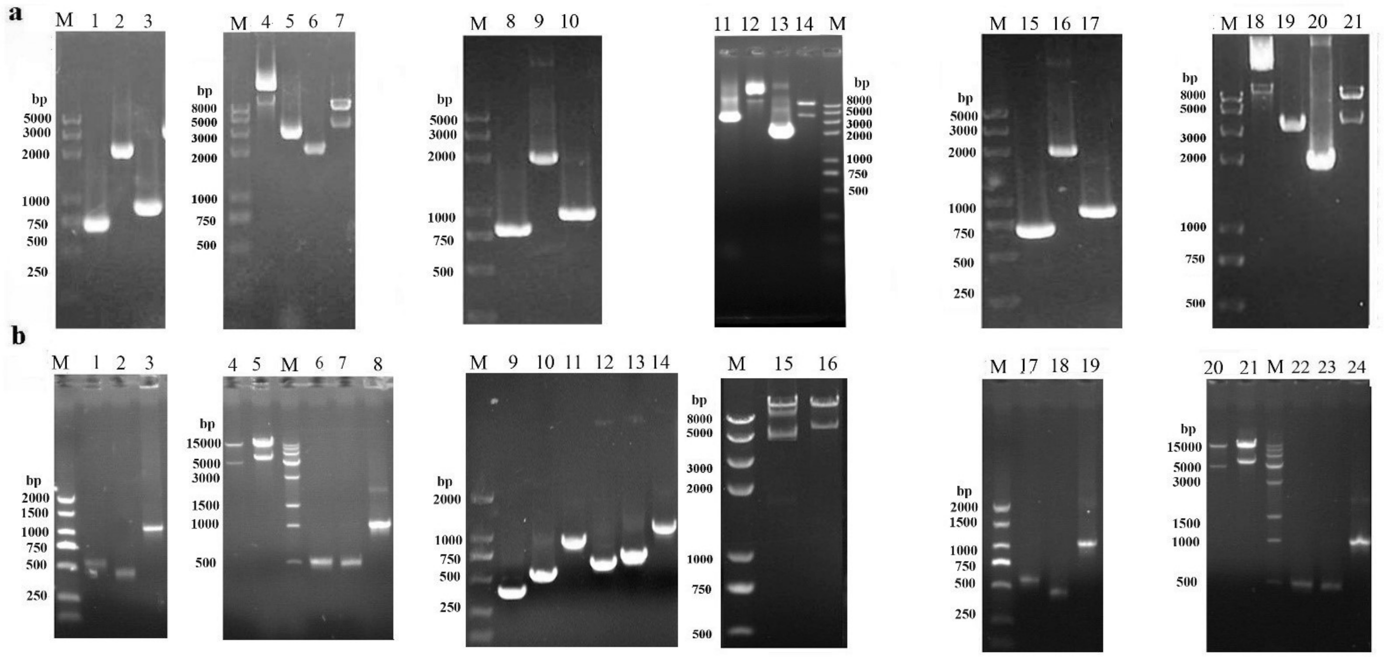


Fig. S1 Nucleic acid electrophoresis of vectors construction by Gibson assembly

a Construction of knockout vectors for target genes in ATMT. Lane 1/8/15: fragment amplified by primer pair gene-5F/5R (upstream for knockout of *mpclr4*, *mpdot1* and *mplig4*, respectively); lane 2/9/16: fragment amplified by primer pair *hph*-F/R using plasmid pSKH as template; lane 3/10/17: fragment amplified by primer pair gene-3F/3R (downstream for knockout of *mpclr4*, *mpdot1* and *mplig4*, respectively); lane 4/12/18: recombinant vector pCtarget extracted from *E. coli* DH5α; lane 5/11/19: knockout cassette amplified by primers gene-5F/3R using recombinant vector as template; lane 6/13/20: fragment amplified by primer pair *hph*-F/R using recombinant vector as template; lane 7/14/21: recombinant vector by double digestion; lane M: DNA marker. b Construction of knockout vectors for target genes in CGES. Lane 1/10/17: fragment amplified by primer pair P*gdpA*-F/gRNA-gene-R; lane 2/9/18: fragment amplified by primer pair gRNA-gene-F/T*trpC*-R; lane 3/11/19: sgRNA transcript amplified by primer pair P*gdpA*-F/T*trpC*-R; lane 4/15/20: vector pFC332 by double digestion; lane 5/16/21: recombinant vector pFCkotarget by double digestion; lane 6/13/22: fragment amplified by primer pair dDNA-gene-5F/5R (upstream donor DNA for knockout of *mpclr4*, *mpdot1* and *mplig4*, respectively); lane 7/12/23: fragment amplified by primer pair dDNA-gene-3F/3R (downstream donor DNA for knockout of *mpclr4*, *mpdot1* and *mplig4*, respectively); lane 8/14/24: donor DNA amplified by primer pair dDNA-gene-5F/3R for knockout of *mpclr4*, *mpdot1* and *mplig4*, respectively; lane M: DNA marker.

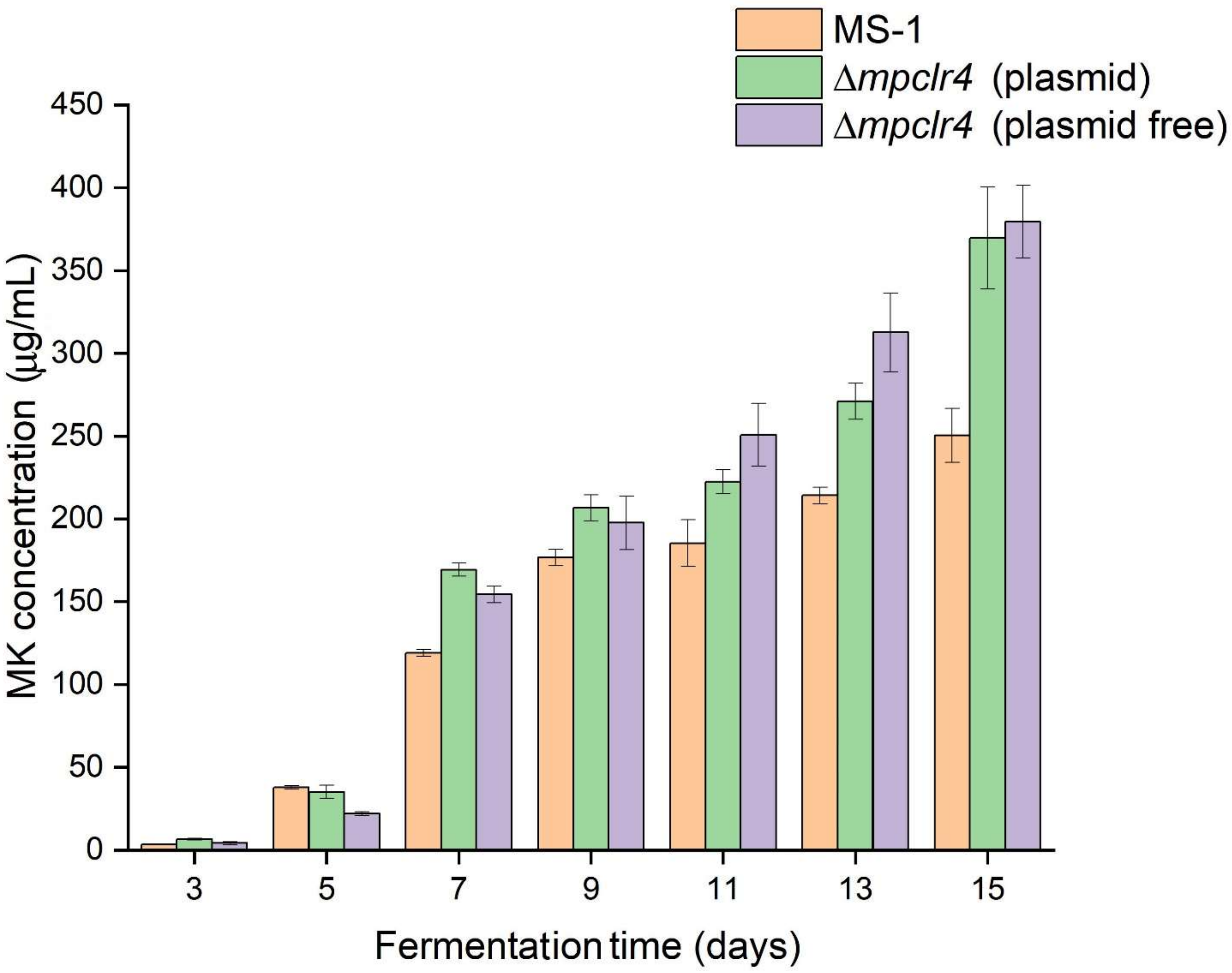


Fig. S2 Yields of MK for strains MS-1, Δ*mpclr4* with plasmid and Δ*mpclr4* without plasmid

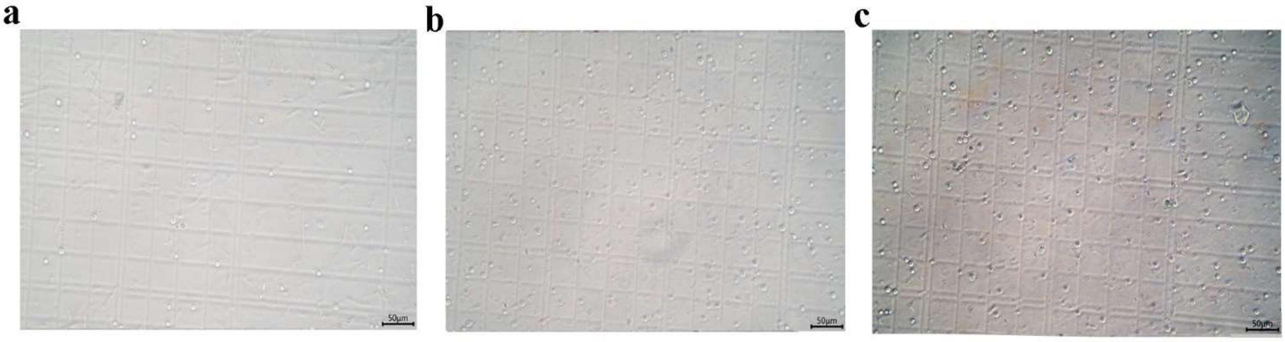


Fig. S3 Protoplasts released by different filamentous fungi

a Protoplasts of *Aspergillus oryzae* released by enzymatic hydrolysis. b Protoplasts of *Monascus pilosus* MS-1 released by enzymatic hydrolysis. c Protoplasts of *Monascus ruber* M7 released by enzymatic hydrolysis.
